# Elucidating salient site-specific functional connectivity features and site-invariant biomarkers in schizophrenia via deep neural networks

**DOI:** 10.1038/s41598-023-48548-w

**Published:** 2023-11-29

**Authors:** Yi Hao Chan, Wei Chee Yew, Qian Hui Chew, Kang Sim, Jagath C. Rajapakse

**Affiliations:** 1 School of Computer Science and Engineering, Nanyang Technological University, Singapore, 639798 Singapore; 2 Research Division, Institute of Mental Health (IMH), Singapore, Singapore; 3 West Region, Institute of Mental Health (IMH), Singapore, Singapore

**Keywords:** Neuroscience, Engineering, Mathematics and computing

## Abstract

Schizophrenia is a highly heterogeneous disorder and salient functional connectivity (FC) features have been observed to vary across study sites, warranting the need for methods that can differentiate between site-invariant FC biomarkers and site-specific salient FC features. We propose a technique named Semi-supervised learning with data HaRmonisation via Encoder-Decoder-classifier (SHRED) to examine these features from resting state functional magnetic resonance imaging scans gathered from four sites. Our approach involves an encoder-decoder-classifier architecture that simultaneously performs data harmonisation and semi-supervised learning (SSL) to deal with site differences and labelling inconsistencies across sites respectively. The minimisation of reconstruction loss from SSL was shown to improve model performance even within small datasets whilst data harmonisation often led to lower model generalisability, which was unaffected using the SHRED technique. We show that our proposed model produces site-invariant biomarkers, most notably the connection between transverse temporal gyrus and paracentral lobule. Site-specific salient FC features were also elucidated, especially implicating the paracentral lobule for our local dataset. Our examination of these salient FC features demonstrates how site-specific features and site-invariant biomarkers can be differentiated, which can deepen our understanding of the neurobiology of schizophrenia.

## Introduction

Schizophrenia is a chronic and debilitating mental illness affecting more than 27 million individuals worldwide^1^. The diagnosis and management of this condition has to take into account the significant heterogeneity in neurobiology^2^ and clinical presentations^3^ which are actively being investigated across sites and clinical settings^4,5^. The examination and discovery of biological factors including neuroimaging biomarkers that are generalisable across sites would promote a better understanding of this disorder and potentially allow for better predictive models, novel treatment targets and prognostication of this condition^6–8^.

One way to extract neuroimaging biomarkers (e.g., from fMRI datasets) is to fit a machine learning model on the datasets and identify the most important features that contributed to the model’s decision. Notably, deep neural networks have been used to model functional connectivity (FC) datasets and have shown better performance than conventional machine learning algorithms such as support vector machine models^9–11^. This suggests that non-linear relationships do exist and warrants the use of more complex models for better elucidation. However, actual implementation of such algorithms is complicated by the high dimensionality of fMRI datasets and coupled with the often smaller datasets available, there is concern about the potential issue of model overfitting on such datasets^12^. To address overfitting, principal component analysis or recursive feature elimination can be used to reduce data dimensionality when training disease classification models^13^. Recently, Gupta et al.^14^ had suggested to address overfitting in deep learning models by removing accessory nodes (that are irrelevant to the prediction) from hidden layers in the neural network. While feasible when the dataset size is larger (e.g., in hundreds), such feature selection or pruning approaches eventually reach a limit when being applied on even smaller datasets from a single site.

Another way to address overfitting is to find other open-source datasets and use it along with the smaller locally collected dataset or through pooling data into collaborative consortiums across multiple sites. In this regard, there are various approaches of pooling datasets together: (i) aggregation without protocol standardisation, i.e. gathering multi-site data into a uniform file format and folder structure (e.g. Autism Brain Imaging Data Exchange^15^, ADHD-200^16^, SchizConnect^17^), (ii) aggregation with protocol standardisation, i.e. carefully designed and enforced data collection protocols that have to be observed at all sites to minimise site differences (e.g. Human Connectome Project^18^, UKBioBank^19^). The latter is ideal since it minimises site differences and labelling inconsistencies, but the process is laborious. We need solutions to better utilise datasets that fall under the former category, consisting of datasets collected over time from different sites with different protocols.

In this regard, SHRED (short for Semi-supervised learning with data HaRmonisation via Encoder-Decoder-classifier)^20^, addresses the issue of site differences and labelling inconsistencies by incorporating both semi-supervised learning (SSL) and data harmonisation in its architecture. This allows as much data as possible to be used for model training. Furthermore, its harmonisation approach—based on a generalisation of ordinary least squares—makes it possible to sieve out site-invariant biomarkers easily, as opposed to extant domain adaptation methods^21^ which cannot produce site-invariant biomarkers as they only map a source domain to a target domain, while existing domain generalisation methods^22^ do not consider the effects of labelling inconsistencies. Thus, SHRED makes it possible to disambiguate biomarkers by separating site-invariant biomarkers from site-specific features. The site-specific salient FC features may indicate neuroimaging features associated with certain subpopulations or subtypes while the site-invariant salient FC features could point to neuroimaging biomarkers generalisable across a wider population.

In this study, we sought to better identify neuroimaging biomarkers in schizophrenia from resting state functional MRI assessments by combining our own dataset with other openly available datasets from SchizConnect^17^ and the UCLA Consortium for Neuropsychiatric Phenomics LA5c Study^23^. In view of the need for better data harmonisation across four sites with differing samples, we used a deep neural network approach based on SHRED^20^ to determine site-specific (unique to local population) salient FC features and site-invariant FC biomarkers (generalisable to wider population) in schizophrenia.

## Methods

### Local clinical sample and external data sets

We recruited local subjects (patients with schizophrenia and age, gender matched healthy controls) from the Institute of Mental Health, Singapore and community respectively after a full explanation of the study procedures and provision of informed consent by all participants. The inclusion criteria were: (1) DSM-IV diagnosis of schizophrenia; (2) Age 21–65 years old; (3) English speaking subjects, and exclusion criteria consisted of: (1) history of significant head injury; (2) neurological diseases such as epilepsy, cerebrovascular accident; (3) DSM alcohol or substance use or dependence; (4) contraindications to MRI (e.g. pacemaker, orbital foreign body, recent surgery/procedure with metallic devices/implants deployed); (5) pregnancy. This study was approved by the Institution Review Boards of Institute of Mental Health and National Healthcare Group (DSRB Reference 2012/01,116). All methods were performed in accordance with the relevant guidelines and regulations.

For the local dataset, single session MRI scans were acquired on a state-of-the-art, clinical 3-Tesla system (Achieva 3T, Philips Medical Systems, Netherlands). Subjects were instructed to remain still and keep their eyes open and fixed on the centre cross of the screen. Whole brain, high resolution, 3D MP-RAGE (magnetisation-prepared rapid acquisition with a gradient echo) volumetric scans (TR/TE/TI/flip angle 8.4/3.8/3000/8; matrix 256 × 204; FOV 240 mm^2^) with axial orientation (reformatted to coronal), covering the whole brain for structural-anatomic detail were acquired.

In addition, data sets from 3 separate sources were used i.e. UCLA Consortium for Neuropsychiatric Phenomics LA5c Study (https://openneuro.org/datasets/ds000030/), COllaborative Informatics and Neuroimaging Suite Data Exchange tool (COINS; http://coins.mrn.org/dx) and Neuromorphometry by Computer Algorithm Chicago (NMorphCH) dataset (http://nunda.northwestern.edu/nunda/data/projects/NMorphCH). Further summarised details of the 4 datasets can be found in the Supplementary Note.

All resting state fMRI scans were pre-processed via fMRIPrep 21.0.1. Details about the pre-processing steps can be found in the Supplementary Note. In brief, pre-processing steps included motion correction, slice timing correction and band-pass filtering. After pre-processing, FC matrices were generated by (i) applying Power’s atlas^24^ to derive 264 regions of interest (ROI), (ii) computing the mean time series by taking the average of all voxels within a 2.5 mm radius from the ROI, (iii) calculating the Pearson’s correlation score between every ROI pair. This produces a 264 × 264 correlation matrix for every subject. Since the matrix is symmetrical, only the bottom triangular is used as input features to SHRED-III by flattening them into a vector as vanilla deep neural networks can only take in vectors as input.

### SHRED architecture

Here in Sections “Harmonising at the encoder input”–“Biomarker generation”, we introduce the SHRED architecture^20^ along with additional improvements which outperformed previous versions of the SHRED model. We named this updated version SHRED-III. Source code for the architecture is available at https://github.com/SCSE-Biomedical-Computing-Group/SHRED.

**Figure 1:**
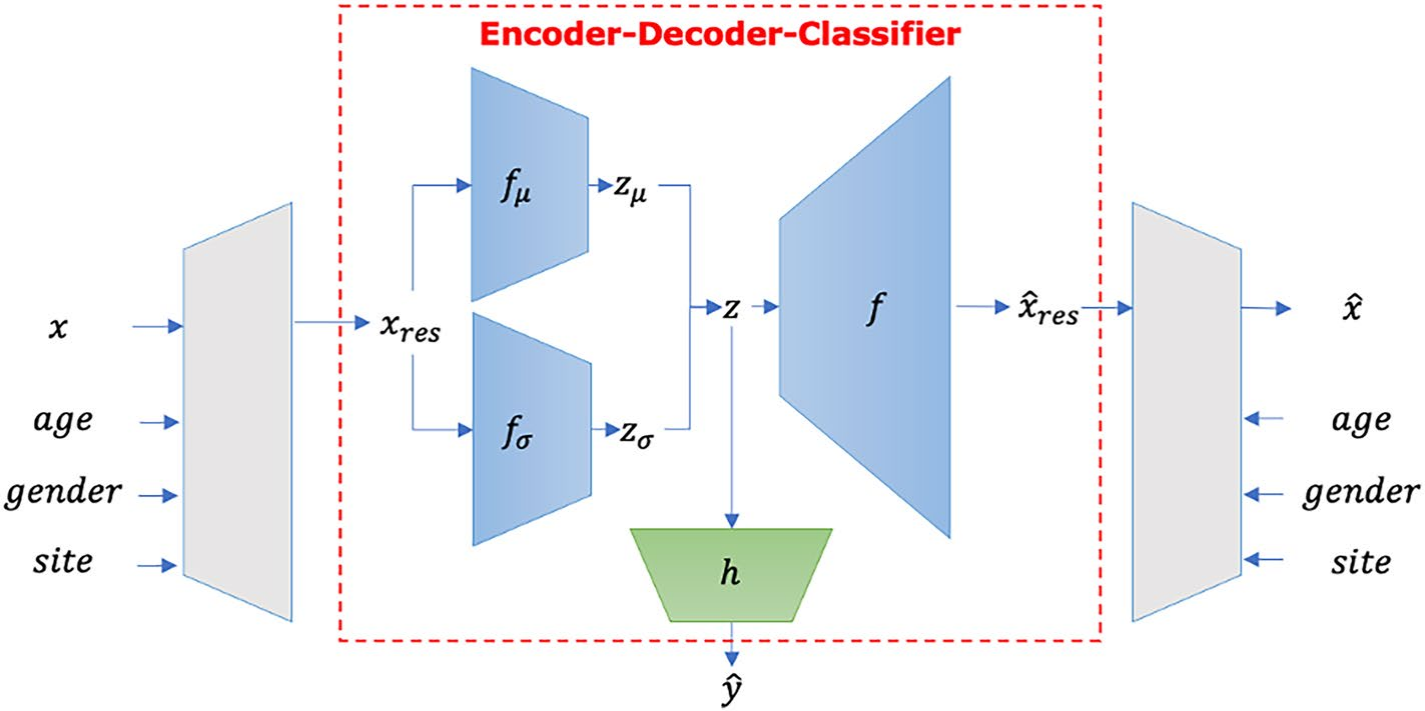
Illustration of SHRED, consisting of a set of harmonisation (grey trapezoids) modules implemented via linear layers and an EDC module implemented via a variational auto-encoder.

As illustrated in Fig. 1 below, SHRED is composed of 3 parts: an encoder-decoder-classifier (EDC) module which permits semi-supervised learning (SSL) and a pair of harmonisation modules, one at each side of the EDC, which performs data harmonisation.

SHRED-III introduces two key changes from SHRED: (i) covariates of interests in the design matrix are not added back (i.e. both site effects and confounders are removed), (ii) the loss function, specifically the formulation of the reconstruction loss, is improved to better approximate the site effects. Additionally, SHRED-III is trained in an end-to-end manner, unlike SHRED-II where ComBat was first applied before the harmonised data is passed to EDC in a two-stage approach.

#### Harmonising at the encoder input

Following the notations in Chan et al.^20^, let $X\in {\mathbb{R}}^{N \times V}$ represent a dataset with $N$ subjects and $V$ functional connectivity (FC) features gathered from $I$ sites. Subjects $S={S}_{I}\cup {S}_{u}$ are made up of a set ${S}_{l}$ of labelled subjects from a single site and a set ${S}_{u}$ of unlabelled subjects from other sites. For a single subject $j\in S$, let ${x}_{j}={{(x}_{jv})}_{v\in V}$ denote its corresponding FC vector.

For a subject $j$ scanned at site $i$, let ${x}_{ijv}$ be the value of FC feature $v$. We model ${x}_{ijv}$ as ${x}_{ijv}={\alpha }_{v}+{M}_{jv}{\beta }_{v}+ {\gamma }_{iv}+{\delta }_{iv}{\varepsilon }_{ijv}$, where ${\alpha }_{v}$ denotes an approximation to the mean value across subjects $S$ of feature $v$, ${M}_{jv}$ denotes a design matrix consisting of covariates of interest (e.g. age, gender), ${\beta }_{v}$ denotes a vector of coefficients corresponding to ${M}_{jv}$, ${\gamma }_{iv}$ and ${\delta }_{iv}$ denotes the additive (or location parameter) and multiplicative (or scale parameter) site effects of site $i$ for feature $v$ respectively, ${\varepsilon }_{ijv}$ denotes a residual term which arises from a normal distribution with zero mean and variance ${\sigma }_{v}^{2}$.

The breakdown of ${x}_{ijv}$ is similar to the formulation in ComBat^25^, but we note that our optimisation process differs (as explained in Section “End-to-end training”) as there is no clear way to integrate its Bayesian-based approach of parameter fitting into deep neural network architectures. Furthermore, our approach of removing site differences allows biomarkers to be easily derived via computing saliency scores^14^ since the implementation is simply based on linear layers.

$\alpha$ is first initialised as a vector of zeros of the same length as the feature vector $v$ and is gradually learnt by the network during model training. Covariates in the design matrix, which are commonly represented as continuous or categorical features, are similarly mapped to a length $V$ vector. The same applies to the site effect terms $\gamma$ and $\delta$ which are initially one-hot encoded and projected by a set of learnable weights in a linear layer. These weights are trained along with the rest of the model, as detailed in Section “End-to-end training”.

Having broken ${x}_{ijv}$ down into its constituent parts, the output of the harmonisation module is then formed by removing site effects and covariates, resulting in ${x}_{res}={\alpha }_{v}+{\varepsilon }_{ijv}$. Here, we emphasise that rather than adopting the common interpretation that ${\varepsilon }_{ijv}$ encodes noise, it is expected that ${\varepsilon }_{ijv}$ captures the idiosyncrasies of feature $v$ in each subject which is not captured by ${\alpha }_{v}$. Overall, this harmonisation step reduces site effects, allowing FC features obtained from multiple sites to be used simultaneously.

#### Encoder-decoder-classifier architecture

The EDC module, which performs SSL, consists of a variational auto-encoder (VAE) with a vanilla deep neural network (DNN) classifier attached at the bottleneck layer of the VAE to perform disease classification. The VAE takes in the output of the harmonisation module ${x}_{res}$ as input and produces a reconstruction ${\widehat{x}}_{res}$ as output. The DNN branch produces $\widehat{y}$ as output, which is then compared against the actual label to improve the model.

The VAE component of the EDC module is made up of two encoders, ${f}_{\mu }$ and ${f}_{\sigma }$. They encode the input ${x}_{res}$ by learning a normal distribution with a mean of ${z}_{\mu }$ and standard deviation of ${z}_{\sigma }$, followed by a sampling procedure that produces the learnt representation $z$. The EDC module also has a decoder $f$ which takes in $z$ and learns to produce a reconstruction of the input data ${x}_{res}$, producing ${\widehat{x}}_{res}=f(z)$. This allows for an incorporation of reconstruction loss to guide model training even in cases where label inconsistency makes it less possible to utilise all labels available in the dataset.

The DNN component of the EDC module takes in the latent representation $z$ from the VAE as input and performs disease classification using it. Letting $h$ denote the DNN mapping, the classification output is denoted by $\widehat{y}={\text{soft}}{\text{max}}(h\left(z\right))$. This provides a means for supervised learning to occur in the model, whilst the model produces its prediction for each data sample. In sum, the VAE and DNN components of the EDC module were used together to help the model generalise better i.e. besides making accurate classifications, it also had to learn representations that were useful enough to allow for accurate reconstruction of the inputs.

#### Harmonising at the decoder output

The VAE in the EDC module has produced ${\widehat{x}}_{res}$, a reconstruction of its input ${x}_{res}$. It has not reconstructed the original input $x$ yet. Evidently, there is still a step remaining to link ${\widehat{x}}_{res}$ to $\widehat{x}$, the reconstruction of the original input of the entire architecture (i.e. before data harmonisation). Since the harmonisation module is used on $x$ to produce ${x}_{res}$, producing $\widehat{x}$ requires the steps taken in the harmonisation module to be reversed. ${\widehat{x}}_{ijv}$, just like ${x}_{ijv}$, can be decomposed into: ${\widehat{x}}_{ijv}={\alpha }_{v}+{M}_{jv}{\beta }_{v}+{\gamma }_{iv}+{\delta }_{iv}{\widehat{\upvarepsilon }}_{\mathrm{ijv}}$ and ${\widehat{\upvarepsilon }}_{\mathrm{ijv}}$ is obtained from ${\widehat{x}}_{res}$ via ${\widehat{\upvarepsilon }}_{\mathrm{ijv}}={\widehat{x}}_{res}-{\alpha }_{v}$. This demonstrates how $\widehat{x}$ is produced, completing the reconstruction.

#### End-to-end training

SHRED-III is trained end-to-end via a single multi-objective loss function where weights are learnt to simultaneously remove site effects and maximise classification performance. Just like how the EDC module was presented, the components of the loss function can be grouped under two categories: the DNN component and the VAE component.

The DNN component of the EDC module makes use of the cross-entropy loss ${L}_{C}$ which compares the output of the classifier $\widehat{y}$ with the ground truth disease labels $y$. This is only computed when labelled data is provided to the model.

$${L}_{C}=\frac{1}{\left|{S}_{l}\right|}\sum_{j\in {S}_{l}}{\sum }_{c=1}^{C}-{y}_{jc} log {\widehat{y}}_{jc}$$

The VAE component of the EDC module, on the other hand, makes use of both labelled and unlabelled data. It consists of three parts: ${L}_{L}$, ${L}_{D}$ and ${L}_{R}$.

First, a likelihood loss ${L}_{L}$ is introduced to maximise the log likelihood of input $x$ being sampled from Gaussian distribution with standard deviation ${\sigma }_{v}$.

$${L}_{L}=NLL\left(x,\widehat{x},\sigma \right)=\frac{1}{2N}{\sum }_{j=1}^{N}{\sum }_{v=1}^{V}\left(log \left(2\pi {\sigma }_{v}^{2}\right) +\frac{{\left({x}_{jv}-{\widehat{x}}_{jv}\right)}^{2}}{{2\sigma }_{v}^{2}}\right)$$

Second, VAE also minimizes the Kullback–Leibler divergence ${L}_{D}$ between the distribution of the latent representations learnt by SHRED-III and a Gaussian prior $p\left(z\right)=N\left(\mathrm{0,1}\right)$. We used ${L}_{D}$ in the form of Evidence Lower Bound (ELBO)^26^.

$${L}_{D}=\frac{1}{2N}{\sum }_{j=1}^{N}{\sum }_{v=1}^{V}\left({{z}_{\sigma }^{2}}_{jv}+{{z}_{\mu }^{2}}_{jv}-2 log {{z}_{\sigma }}_{jv}-1\right)$$

Third, for accurate reconstruction of input data $x$ by the VAE, reconstruction loss ${L}_{R}$ is used with an objective to maximize the log likelihood of input ${x}_{ijv}$ being sampled from a Gaussian distribution $N\left({\alpha }_{v},{\sigma }_{v}^{2}\right)$ where ${\sigma }_{v}^{2}$ represents the variance of feature v across subjects. Also, we maximise the log likelihood of ${x}_{ijv}-{\alpha }_{v}$ being sampled from another Gaussian distribution $N\left({\gamma }_{iv},{\delta }_{iv}^{2}\right)$. These newly proposed changes from SHRED^20^ (which originally minimised ${\varepsilon }_{jv}$ and the difference between ${\alpha }_{v}+{M}_{jv}{\beta }_{v}$ and the mean feature vector) are designed to provide a closer approximation to the optimisation process used in ComBat^25^, where the site effects were estimated via empirical Bayes (SHRED did not consider them). Denoting ${\uptheta }_{\mathrm{ijv}}$ as ${x}_{\mathrm{ijv}}-{\alpha }_{v}$, we have:

$${L}_{R}=\frac{1}{2N}{\sum }_{j=1}^{N}{\sum }_{v=1}^{V}\frac{1}{2}\left(log \left(2\pi {\sigma }_{v}^{2}\right) +\frac{{\left({\uptheta }_{\mathrm{ijv}}\right)}^{2}}{{2\sigma }_{v}^{2}}+log \left(2\pi {\delta }_{iv}^{2}\right) +\frac{{\left({\uptheta }_{\mathrm{ijv}}-{\upgamma }_{\mathrm{iv}}\right)}^{2}}{{2\delta }_{iv}^{2}}\right)$$

All 4 loss terms are then combined, forming a joint loss $L= {L}_{C}+{\gamma }_{1}{L}_{L}+{\gamma }_{2}{L}_{D}+{\gamma }_{3}{L}_{R}$ where ${\gamma }_{1}$, ${\gamma }_{2}$ and ${\gamma }_{3}$ are hyperparameters tuned during model training. $L$ is minimised using the Adam optimiser.

#### Biomarker generation

Once a model is trained, feature attribution scores are computed from the output layer to input layer. In this work, we use Integrated Gradients^27^ to generate the scores. Many of these feature attribution methods are equivalent, especially when the model is simple (i.e., close to linear) but amongst them, Integrated Gradients remains usable for a wider variety of situations^28^. Along with the clear choice of baseline to use (average of normal controls’ input data), these reasons explain the rationale behind our choice of using Integrated Gradients.

Let $F$ represent the model to be decoded. Feature attribution scores can then be computed using Integrated Gradients via the following expression:

$${R}_{v}({x}_{j}|F)=\left({x}_{jv}-\overline{x }\right){\int }_{\mathrm{a}=0}^{1}\frac{\partial F \left(\overline{x }-a\left({x}_{j}-\overline{x }\right)\right)}{\partial {x}_{jv}}da$$

where $\overline{x }$ represents the baseline and ${R}_{v}({x}_{j}|F)$ represents the feature attribution score for feature $v$, given input data ${x}_{j}$. The baseline is computed by taking the mean of all normal controls in the training dataset.

These scores provide a quantitative measure of each feature’s contribution to the task. Notably, these are computed for individual samples. This means that feature attribution scores computed for each sample can be used to generate individualised biomarker heatmaps, allowing for personalised treatments to be designed.

Group-level studies (case–control) are performed too by aggregating the feature attribution scores:

$${R}_{{v}_{F}}=\frac{1}{M}{\sum }_{j\in \{j|{y}_{j}=1\}}\left|{R}_{v}({x}_{j}|F)\right|$$

${R}_{{v}_{F}}$ represents the group-level feature attribution score which is obtained by taking the average feature attribution score of $M$ test subjects. These scores are then used to produce biomarker heatmaps presented in Figs. 3 and 4.

### Experiment setup

In this study, we are interested to elucidate (i) site-invariant FC biomarkers and (ii) site-specific salient FC features. Thus, two sets of experiments were conducted.

1. Whole dataset

To arrive at site-invariant FC biomarkers, data from multiple sites are used simultaneously. We aim to demonstrate that SHRED-III is capable of producing site-invariant biomarkers even when the whole dataset is used. This is in contrast to existing approaches that use supervised learning models on the whole dataset, which we hypothesise will produce biomarkers that are biased towards the larger sites.

SHRED-III involves both data harmonisation and SSL being used simultaneously. To study how each component of SHRED-III contributes to the model performance, an ablation study was conducted. This involves DNN (no harmonisation, no SSL), DNN (DH) (harmonisation but no SSL). EDC (SSL but no harmonisation), SHRED-III (harmonisation and SSL). Since the whole dataset is used, SSL does not incorporate the idea of using unlabelled data in this setting—rather, it is the use of the reconstruction loss that is being analysed. We also conducted experiments with SHRED and SHRED-II to compare with approaches proposed in previous studies.

2. Individual sites

To arrive at site-specific salient FC features, models were fitted to individual sites separately. This involved four experiment settings: (i) Supervised learning (SL), (ii) SL + data harmonisation, (iii) SSL, (iv) SSL + data harmonisation. For SL, two architectures were used: vanilla DNN and EDC (without unlabelled data). For SSL, EDC (with unlabelled data) and 3 SHRED variants (SHRED, SHRED-II and SHRED-III) were used.

Hyperparameters used in these architectures were tuned following the approach mentioned in Chan et al.^20^. In brief, tuning was performed on a subset of the ABIDE I dataset which was not used for the main set of experiments. There are several reasons supporting this decision: (i) given the relatively small dataset size in this work (around 400, as compared to 700–800 used previously), we do not want to tune and overfit to this dataset, (ii) all 3 datasets are based on FC data and the obtained hyperparameters were already shown to generalise well to the ADHD-200 dataset in the previous work, thus it is reasonable to expect that they will work for these schizophrenia datasets as well, (iii) tuning specifically to the Schizophrenia dataset will leave too little data for model testing and we want to run our models over multiple seeds and folds in a cross-validation approach so as to demonstrate the robustness of our obtained biomarkers.

The following parameters were tuned for EDC: number of hidden layers in the DNN branch $\{\mathrm{0,1},2\}$, size of hidden layer in the VAE branch $\{\mathrm{32,64,128}\}$, size of latent representation in VAE $\{\mathrm{16,32,64}\}$, dropout $\left\{0.0, 0.1, 0.2, 0.3\right\}$, ${\gamma }_{1} \{{10}^{-5},{10}^{-4},\dots , 1\}$, ${\gamma }_{2} \{{10}^{-4},{10}^{-3},{10}^{-2}\}$, learning rate $\{0.00025, 0.0005, 0.001, 0.002, 0.004\}$. For the SHRED variants, we further tuned ${\gamma }_{3} \{{10}^{-5},{10}^{-4},\dots , 1\}$ and while doing so, tuned ${\gamma }_{1}$ and ${\gamma }_{2}$ again. 300 iterations of Bayesian optimization were used for tuning.

The final architecture for EDC includes: a single Dense layer for the classifier (with two output neurons for binary disease classification), 32 hidden neurons in the encoders ${f}_{\mu }$ and ${f}_{\sigma }$ as well as in the decoder $f$, 16 neurons in the latent representation of the VAE, dropout of 0.2. The hyperparameters ${\gamma }_{1},{\gamma }_{2}$ were set to ${10}^{-4},{10}^{-3}$. For SHRED-I, ${\gamma }_{3}$ was set to ${10}^{-0}$.

For SHRED-III ${\gamma }_{1},{\gamma }_{2}$ and ${\gamma }_{3}$ were set to ${10}^{-5},{10}^{-3}$ and ${10}^{-4}$ respectively. Adam optimizer with a learning rate of 0.002 and weight decay of 0.001 was used to train all the models. For DNN, the number of neurons were controlled such that the number of parameters used are similar to the other architectures. This resulted in a neural network with 3 hidden layers (with 75, 50, 30 hidden nodes respectively).

## Results

In this section, we provide details about the datasets used and present results in terms of model performance and biomarkers obtained via SHRED-III.

### Datasets

Four relevant resting state fMRI datasets were included in this study, including a local dataset (IMH). Table 1 presents a summary of the demographic features seen in the 4 datasets.

**Table 1:** Demographic features of the 4 datasets included in this study.

Dataset	COBRE	IMH	NMorphCH	UCLA
Subject (NC/SZ)	75/60	18/20	35/38	121/50
Gender (F/M)	34/101	18/20	29/44	68/103
Age range	18–66	23–58	19–46	21–50
PANSS (total)	–	40.2 $\pm$ 6.9		

*PANSS* positive and negative syndrome scale, for schizophrenia patients.

Overall, 417 resting state fMRI scans comprising of 249 normal controls (NC) and 168 schizophrenia patients (SZ) were included from 4 sites. PANSS scores are only available for our local dataset and we note that these patients have a relatively low Positive and Negative Syndrome Scale (PANSS) score, indicating that most have mild symptoms (PANSS scores ranging from 30 to 53).

**Figure 2:**
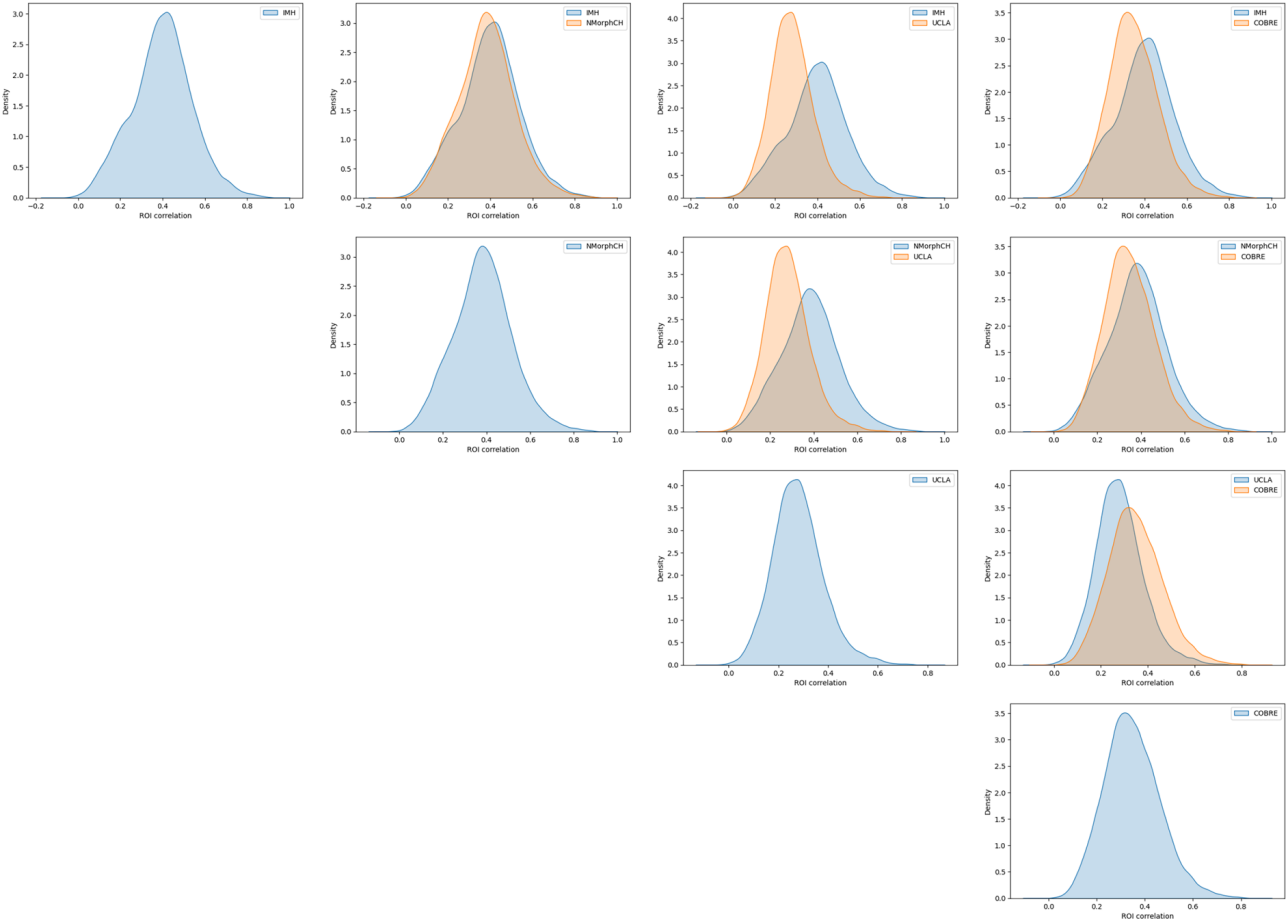
Kernel Distribution Estimation plots of the dataset distribution from each site.

Site differences in the 4 datasets are shown in Fig. 2. These plots were generated by computing the average Fisher-transformed functional connectivity scores and revealed significant distribution differences between each pair of datasets, except for NMorphCH and IMH. Overall, it is evident that despite sharing some similarities (use of same clinical classification DSM-IV), there are significant differences in terms of both data distribution as well as the criteria used for determining class labels for each subject. This suggests the need for methods, such as SHRED-III, to address them. Figure S1 provides quantitative measures (via Hellinger distance) and visualisations of how data harmonisation techniques like ComBat and SHRED reduced site differences. However, it is unclear how these harmonisation techniques affect biomarker discovery. The following sub-sections detail the experiments conducted to investigate the impact of addressing site differences and labelling inconsistencies.

### Comparison of performance with existing baselines

All experiments were performed with fivefold cross-validation repeated across 10 seeds. Tables 2 and 3 show the classification accuracies for all tested model configurations for each individual site and the whole dataset, respectively. All *p*-values reported below were computed based on Welch t-test (two-sample t-test with unequal variances).

**Table 2:** Classification accuracies and standard deviations of the 5 model architectures on individual sites.

	SL	DH + SL	SSL	DH + SSL		
	DNN	DNN	EDC	SHRED	SHRED-II	SHRED-III
COBRE	77.78 ± 2.26	78.52 ± 1.35	82.00 ± 0.78	75.78 ± 1.88	82.15 ± 1.23	76.89 ± 3.85
IMH	61.61 ± 4.07	57.96 ± 2.97	77.93 ± 4.67	73.71 ± 4.55	69.96 ± 2.96	79.18 ± 3.49
NMorphCH	66.01 ± 3.54	63.40 ± 4.07	75.58 ± 2.02	73.02 ± 1.96	75.35 ± 1.81	71.36 ± 4.97
UCLA	79.48 ± 1.34	81.05 ± 1.74	83.84 ± 1.39	83.56 ± 1.05	83.74 ± 1.23	83.92 ± 1.19

*SL* supervised learning, *DH* data harmonisation, *SSL* semi-supervised learning.

**Table 3:** Classification accuracies and standard deviations of the 5 model architectures on the whole dataset (i.e. only SL).

	DNN	DNN (DH)	EDC	SHRED	SHRED-II	SHRED-III
Whole dataset	76.61 ± 1.05	73.46 ± 0.94	76.63 ± 0.54	71.04 ± 1.42	74.15 ± 0.78	75.24 ± 2.09

When data harmonisation was introduced to the vanilla DNN (Table 2), the changes in classification accuracy for individual sites were largely not significant ($P=.04$ for UCLA, $P>.05$ for the other sites). A similar finding was observed when comparing EDC to SHRED-II (which is equivalent to EDC (DH)), with differences in accuracies not significant except for IMH where the accuracy dropped significantly ($P=5\times {10}^{-4})$. On the other hand, in the whole dataset setting (Table 3), a statistically significant decrease in model performance happened when data harmonisation was introduced to both DNN (comparison between DNN and DNN (DH), $P=1\times {10}^{-5}$) and EDC (comparison between EDC and SHRED-II, $P= 6\times {10}^{-4}$). Notably, SHRED-III did not experience as much of a decrease as SHRED and there was no significant change in its performance with respect to EDC $(P=.12)$ and DNN ($P=.12$).

The introduction of SSL brought about a statistically significant increase in model performance ($P< .005$) for all 4 sites as seen in Table 2 (DNN (SL) vs EDC (SSL)). This increase could be due to two possible factors: the use of reconstruction loss to improve latent representations learnt by the model, or the introduction of additional unlabelled data (which also contributes via the reconstruction loss, but in the former only data from the site is used). The results in Supplementary Table S1 for EDC (SL), which combines both cross entropy loss and reconstruction loss but does not use additional unlabelled data from other sites, suggest that the improvements seen in Table 2 largely stem from the introduction of reconstruction loss. Furthermore, the difference between EDC (SL) and EDC (SSL) was largely statistically insignificant ($P>.49$, except for the case of IMH $P=.059$). One possible explanation for this is that the IMH dataset is the smallest (n = 38) and stands to gain more from the unlabelled data (which is about 10 times larger) as compared to the other sites.

Finally, the combination of data harmonisation and SSL can be studied by analysing the results from the variants of SHRED. Comparing EDC with SHRED-II in Table 2 shows that the addition of ComBat did not have much effect on model performance, except for the case for the IMH data ($P=5\times {10}^{-4}$) where performance decreased. From the experiments on the whole dataset, model performance of DNN, EDC and SHRED-III were very similar and the differences were not statistically significant ($P>.1$) between DNN/EDC vs SHRED-III).

From Tables 2 and 3, it seems that the main statistically significant improvement in classification accuracies occurred when SSL was introduced. Harmonisation did not seem to cause significant changes in model performances especially for individual sites. However, sensitivity and specificity values shown in Supplementary Tables S2–S5 provide a clearer picture about the effects of data harmonisation. For individual sites (Supplementary Tables S2, S4), data harmonisation clearly led to a decrease in specificity especially for smaller sites (IMH, NMorphCH) for both DNN and EDC. It also led to an increase in sensitivity for individual sites (with the exception of IMH for EDC). On the other hand, experiments on the whole dataset (Supplementary Tables S3, S5) revealed the opposite pattern: data harmonisation increased specificity, but decreased sensitivity. The extent of the decrease is larger than the increase in specificity, suggesting that the decrease in accuracy seen in Table 3 could be due to fewer true positives being predicted (as a result of data harmonisation).

Additionally, these 4 tables provide more insights into how SSL helped model performance. For individual sites, the specificity values were exceptionally low for IMH and NMorphCH (which are the smaller datasets) when DNN was used, but got much higher when SSL was introduced. Sensitivity generally reduced except for UCLA—this could be explained by how UCLA was relatively imbalanced as compared to other sites. Finally, we note that SHRED-III generally had the highest specificity for both individual sites (except COBRE, but the difference from EDC is not significant) and whole dataset. It also experienced a smaller decrease in specificity (for individual sites) and sensitivity (for whole dataset) due to data harmonisation, as compared to other SHRED models.

Overall, most models were able to perform disease classification rather well. This gives greater confidence to the biomarker heatmaps illustrated in the next section.

### Analysis of salient FC features

To ensure that the salient FC features generated via our approach are robust, saliency scores were averaged across all 50 trained models (from 10 seeds × 5 folds) and standardised. Since the atlas from Power et al.^24^ does not provide anatomical labels, the ROIs were mapped to the labels in Crossley et al.^29^ by matching the closest ROIs in the Crossley atlas to those in the Power atlas, with closeness computed based on Euclidean distance.

Features were generated for two scenarios: on individual sites and on the whole dataset. Features generated from individual sites using models trained via SL will represent site-specific salient FC features, while the features generated from the whole dataset require further examination. It is hypothesised that in the whole dataset setting, (i) when SL models are used, they will produce salient features that are biased towards the larger datasets, (ii) when SHRED-III is used, site-invariant biomarkers will be produced, since site effects and labelling inconsistencies were removed.

#### Site-invariant salient FC features

**Figure 3:**
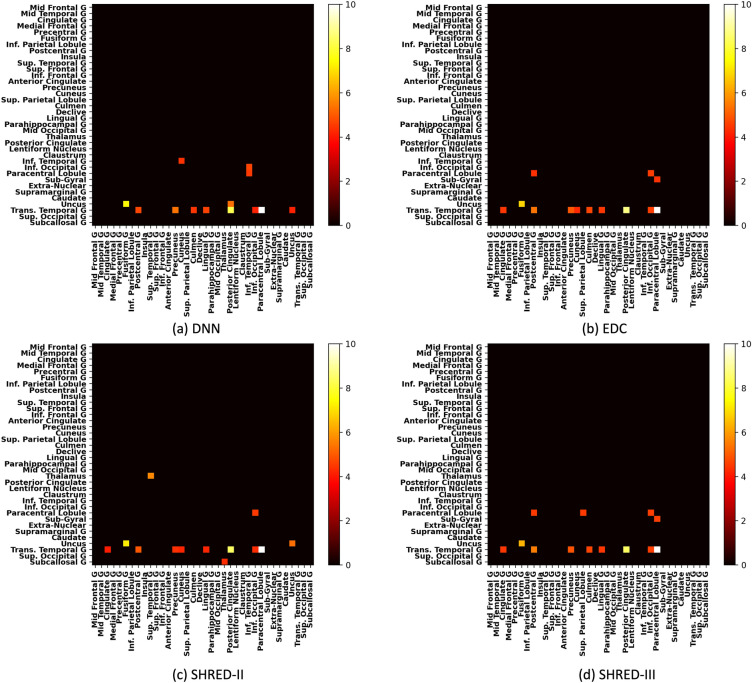
Filtered heatmaps (top 1% features) of saliency scores generated from various models for the whole dataset.

Figure 3 shows the salient FC features from various model configurations in the whole dataset shown in Table 3. For ease of comparison across heatmaps, scores in the upper triangular were set to 0 since the matrix is symmetric. Out of the remaining scores in the lower triangular, only the top 1% features were kept (scores below this threshold were set to 0 for the visualisation). Unfiltered heatmaps are presented in Fig. S3 in the supplementary materials.

From Fig. 3a, the heatmap produced by the vanilla DNN model highlighted 3 potential biomarkers of schizophrenia: transverse temporal gyrus—paracentral lobule, transverse temporal gyrus—posterior cingulate and uncus—fusiform gyrus. These 3 salient FC features are also present in Fig. 3b which was generated from the EDC model. Two notable differences are: (i) a slight decrease in the saliency score for the biomarker uncus-fusiform gyrus, (ii) a decrease in saliency scores across all ROIs (seen from Supplementary Fig. S3b), except for transverse temporal gyrus. When ComBat was introduced to EDC (i.e., SHRED-II), the corresponding heatmap in Fig. 3c shows another three notable differences: (i) another overall decrease in saliency scores relative to EDC (i.e. even greater decrease from DNN as compared to EDC), (ii) increase in saliency scores across multiple ROIs’ connection with thalamus, notably thalamus-superior temporal gyrus, (iii) the connections within uncus has also emerged as a potential biomarker. Notably, these findings were also observed when comparing DNN (Fig. 3a) to the heatmap from DNN + ComBat (Supplementary Fig. S2). Figure 3d shows the heatmap produced by SHRED-III. The most notable change is the further reduction of the saliency scores of the uncus-fusiform gyrus feature.

#### Site-specific salient FC features

**Figure 4:**
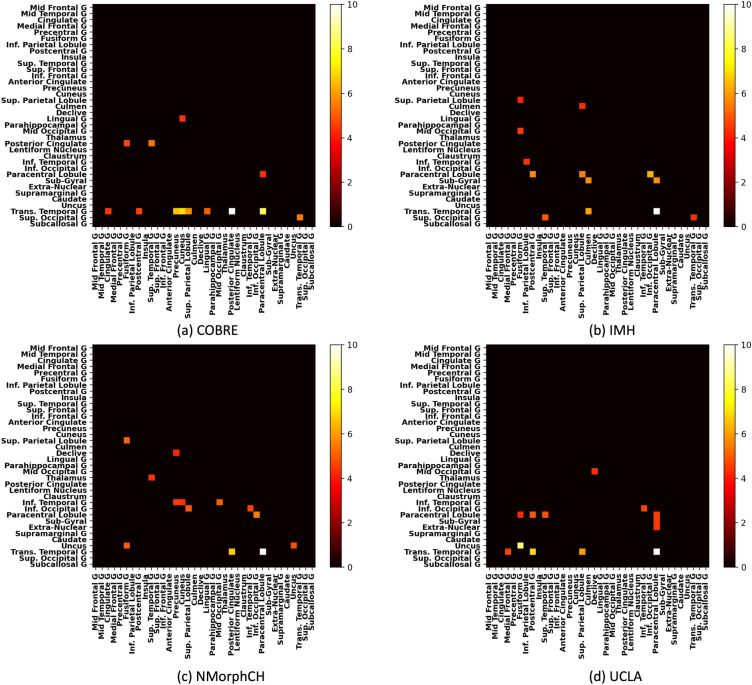
Filtered heatmaps (top 1% features) of saliency scores generated from EDC (SL) for individual sites.

The model from EDC (SL) were used for this as they gave high model performance across all sites, as seen in Supplementary Table S1. Figure 4 shows the site-specific salient FC features generated from EDC (SL) and unfiltered heatmaps are presented in Fig. S4 in the supplementary materials. Figure 4a shows the features obtained from training on the COBRE dataset. Compared to the case where EDC was used on the whole dataset (i.e., Fig. 3b), transverse temporal gyrus emerges as an even more salient feature. Notable changes include the reduction in saliency score for the connection with the paracentral lobule (now eclipsed by the posterior cingulate), along with salient connections with 5 new ROIs: superior parietal lobule, lingual gyrus, superior occipital gyrus and especially precuneus and cuneus. Additionally, uncus—fusiform gyrus, which was previously highlighted as a salient feature in Fig. 3a, b, is no longer salient in Fig. 4a.

Figure 4b shows that the salient FC features obtained from the IMH dataset are quite different. Out of the 3 salient features from Fig. 3b, only transverse temporal gyrus—paracentral lobule remains. It is also the most salient feature for this site. Additionally, paracentral lobule has a noticeably greater contribution: several new salient FC features stem from the connection between paracentral lobule and inferior occipital gyrus, subgyral, postcentral gyrus and superior parietal lobule. Additionally, the connections between culmen with subgyral as well as transverse temporal gyrus are highlighted, along with superior occipital gyrus—superior temporal gyrus.

Salient FC features from NMorphCH bears some similarities with a subset of salient features that are found in COBRE and IMH. There does not seem to be any significant salient features that are specific to NMorphCH. Only 2 salient features from EDC (SL) (whole dataset) remained: transverse temporal gyrus—paracentral lobule and transverse temporal gyrus—posterior cingulate (but the latter has a lower saliency score than before). Notably, transverse temporal gyrus—posterior cingulate was present in COBRE but not IMH. NMorphCH also shares a common salient feature with IMH (paracentral lobule—inferior occipital gyrus).

Finally, salient FC features from UCLA are shown in Fig. 4d. Comparing it to Fig. 3b, transverse temporal gyrus—paracentral lobule was again the most salient feature and uncus-fusiform gyrus is noticeably more prominent here than in other sites. There are two salient features: the connections between transverse temporal gyrus and postcentral gyrus (unique to UCLA), along with the superior parietal lobule (shared with COBRE).

Overall, it can be observed from Fig. 4 that despite site differences, the neuroimaging region of transverse temporal gyrus—paracentral lobule was found to be present across all sites and NMorphCH shares a small subset of salient FC features with IMH.

#### Comparison of group differences in saliency and FC

**Figure 5:**
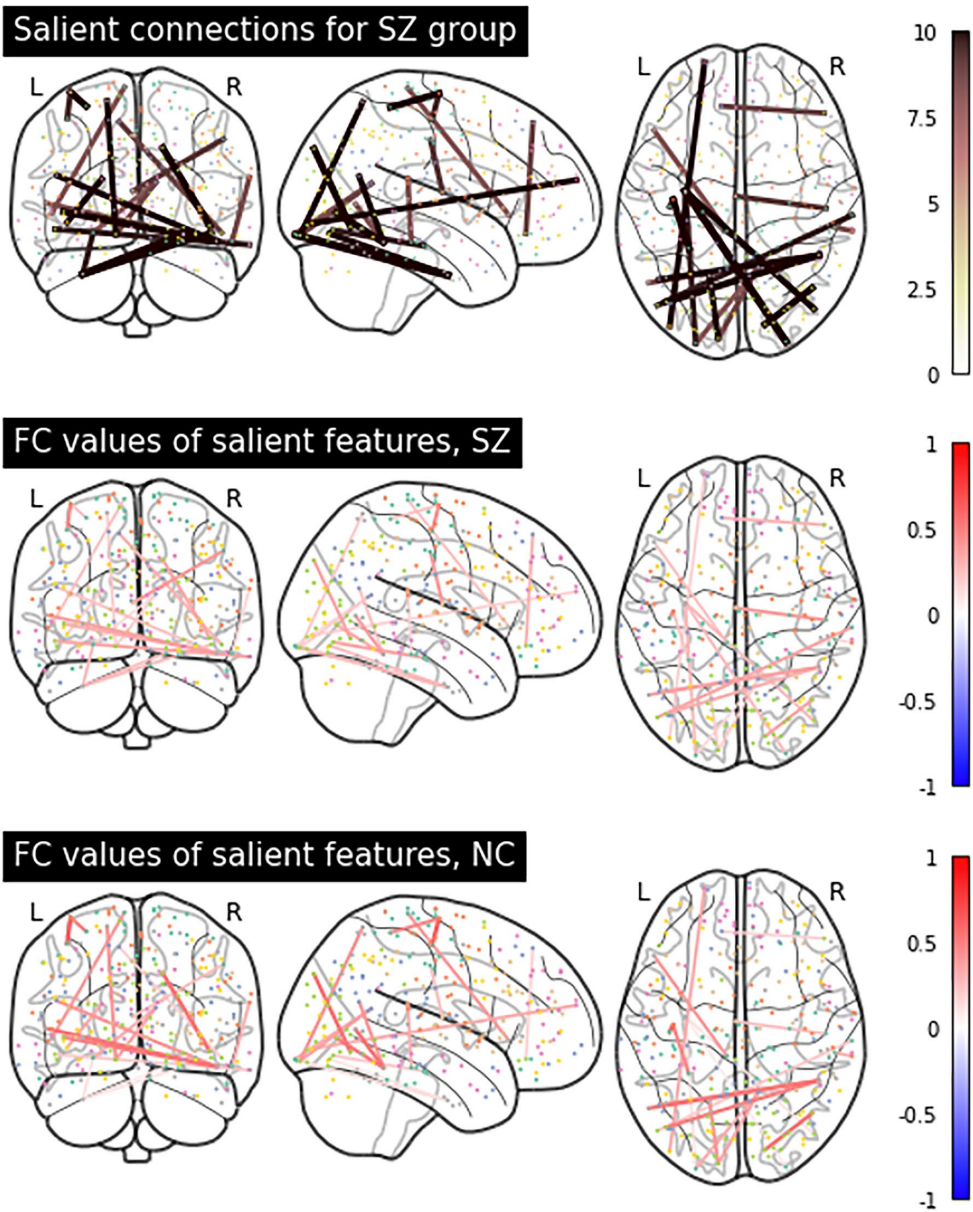
Connectome plots of the top 0.05% salient features’ saliency scores (top row) and original group-level, Fisher-transformed FC values for SZ (middle row) and NC (bottom row).

The heatmaps presented in Figs. 3 and 4 highlighted several salient FC features. To further understand how exactly FC values change in the presence of Schizophrenia, the original FC values of these salient FC features can be visualised separately for each group. Saliency scores generated from the SHRED-III model in the whole dataset setting were used in this analysis. Figure 5 illustrates that in majority of these features, the SZ patients have lower FC values than the NC group. This is especially so for the features with the highest saliency scores, including features previously highlighted such as Uncus—Fusiform Gyrus. Figure S5 provides an alternative visualisation of the salient FC features for SZ via a chord diagram implemented via NiChord^30^, showing the brain modules they belong to.

#### Association study with PANSS scores

**Figure 6:**
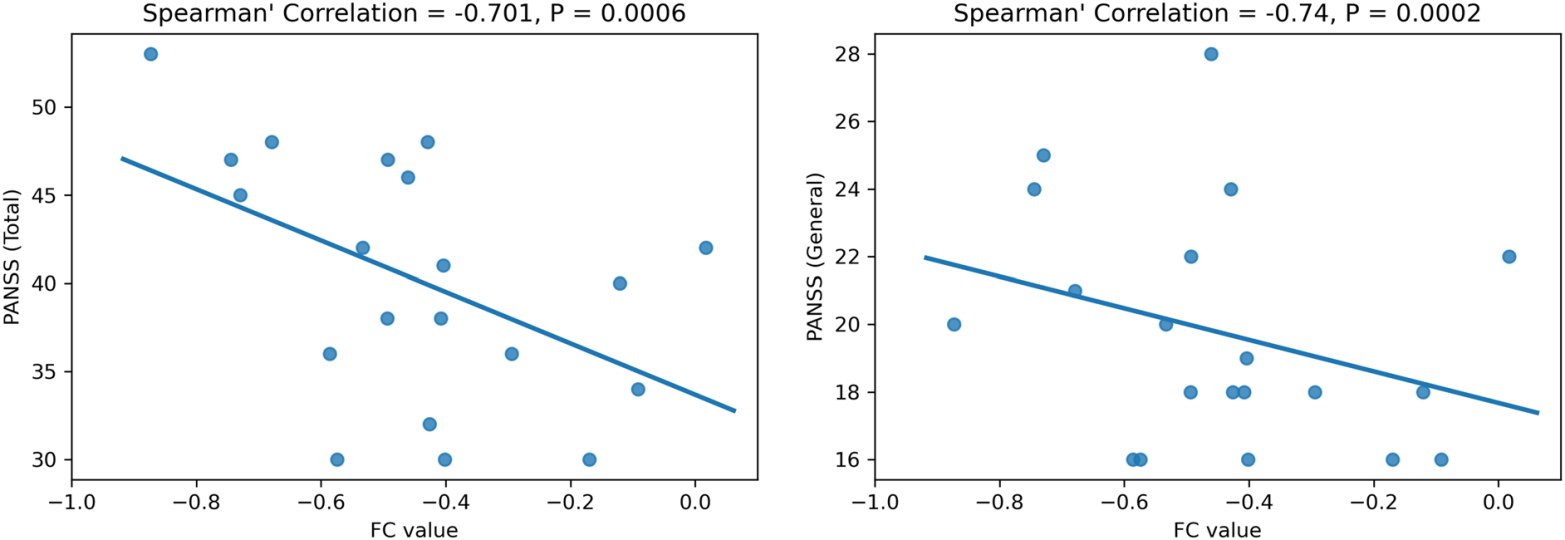
Scatter plots (with regression line) of the FC value from the connection between inferior parietal lobule and parahippocampal gyrus, plotted against PANSS scores.

FC values of salient features can also be correlated with measures of symptom severity of the disorder, such as PANSS for Schizophrenia. To study such associations, FC values of the top 10 salient ROI pairs are correlated with the total PANSS scores, as well as the scores for each subdomain (positive, negative, psychopathology). After corrections for multiple comparisons via the Benjaminini-Yekutieli false discovery rate control procedure, we found that the connection between inferior parietal lobule and parahippocampal gyrus has a significantly strong negative correlation with both total PANSS score (Spearman correlation of − 0.701, $P=6\times {10}^{-4}$) and the general psychopathology scale (Spearman correlation of − 0.74, $P=2\times {10}^{-4}$). Figure 6 illustrates these relationships via scatterplots of FC values and PANSS scores. However, a major caveat to this analysis is the small dataset used—only our local dataset had PANSS scores available. Thus, this only serves as a proof of concept and further studies on bigger fMRI datasets with PANSS scores will be required to validate such associations.

## Summary of the most salient FC features and ROIs

Table 4 summarises the most salient FC features found in this study, presented along with the module that it is associated with as suggested by Power et al^24^. It is evident that majority of the ROIs involve the auditory, visual and sensory/somatomotor networks.

**Table 4:** Summary of the most salient FC features across sites.

ROI 1	System	ROI 2	System	Type
TTG	Auditory	PL	Motor	Site-invariant
TTG	Auditory	PC	DMN	Multiple sites
TTG	Auditory	Cuneus	Visual	Site-specific (COBRE)
PL	Motor	IOG	Visual	Site-specific (IMH)
SPL	Motor	FG	Visual	Site-specific (NMorphCH)
TTG	Auditory	PG	Motor	Site-specific (UCLA)

*TTG* transverse temporal gyrus, *PL* paracentral lobule, *PC* posterior cingulate, *FG* fusiform gyrus, *IOG* inferior occipital gyrus, *SPL* superior parietal lobule, *PG* postcentral gyrus, *DMN* default mode network, *Motor* Sensory/Somatomotor.

Notably, all these features have large positive saliency scores generated from Integrated Gradients. Although only the absolute value of the score was taken (as explained in Section “Biomarker generation”), we found that the most positive saliency scores are always larger in magnitude than the most negative saliency scores. This implies that when the FC value for the corresponding ROI pair shifts away from the baseline (average FC value from that ROI pair for healthy subjects), the model is more likely to predict that schizophrenia is present.

### Discussion

There are several main findings from this study. First, the reconstruction loss in SSL was the key driver of improvement in model performance, while data harmonisation led to reduced model performance that was mitigated when SHRED-III was used. Second, the FC feature transverse temporal gyrus-paracentral lobule was found to be a site-invariant biomarker, as marked by the highest saliency score given within SHRED-III. Third, most sites possess site-specific salient FC features that would not have been captured by existing approaches that work on pooled datasets. For example, paracentral lobule was a salient site-specific feature within the local IMH dataset.

In terms of model performance, our experiments revealed that SSL (specifically the reconstruction loss) is clearly the main contributor to improvements in model performance. This suggests that it is possible to train deep learning models on individual sites and small datasets (< 100 samples) to produce site-specific salient FC features. However, an EDC-based architecture should be used instead of a vanilla DNN (i.e. reconstruction loss should be used) since the specificity of vanilla DNN on small sites was relatively low. It is also evident that data harmonisation caused a slight decrease in model accuracies. Notably, one trend in recent works is to pool datasets from multiple sites together (i.e. whole dataset setting) followed by analysis of the harmonised data. Our results suggested that models trained on such datasets could have poorer generalisability and this was attributed to a large decrease in model sensitivity that outweighed the increase in specificity (i.e. while the model seemed to classify better healthy controls after data harmonisation, it made even fewer correct predictions on cases of illness). Thus, data harmonisation should be used with care and our results suggested that SHRED-III was less susceptible to this problem (much less than SHRED) but there is still some room for improvements to reduce the extent that sensitivity is lowered by data harmonisation.

Another example of a scenario where data harmonisation should be used with care can be seen from the local dataset results. At the level of individual sites, the results from Table 2 showed that data harmonisation led to lower performances on the IMH dataset. Table C.1 further supported this finding across 3 sets of comparisons: DNN, EDC (SL) and EDC (SSL). One reason could be how ComBat has a tendency of imposing greater penalty on smaller sites if there were large dataset size differences across sites^31^. Another possible explanation could be derived from observing the heatmap of the IMH site in Fig. 4b. It is evident that this site has many more site-specific salient FC features (linked to paracentral lobule and sub-gyral) than the other 3 sites. Thus, the use of data harmonisation to remove site difference led to a greater impact on this site than the other sites. Furthermore, discovery of site-specific salient FC features should not be done on datasets that were harmonised. EDC (SL) was shown to be the best approach for such use cases out of the methods analysed in this study.

Overall, combining data harmonisation and SSL via the SHRED variants generally led to better performance than baseline DNN models. However, the combination did not provide as much improvements seen in an early study by Chan et al.^20^ on the ABIDE and ADHD-200 datasets. Thus, our results on this smaller dataset suggested that it might be necessary to collate adequate data from multiple sites (around 800 subjects, based on the size of the ADHD-200 dataset) before the benefits (in terms of model performance) of combining data harmonisation with SSL could be fully observed. One notable phenomenon when training VAE models in small but high dimensional datasets is the possibility of the loss exploding to infinity. In our study, this was resolved by introducing gradient clipping, but we noted that this was not performed in study by Chan et al.^20^ whereby the datasets were larger. Despite these issues, the proposed SHRED-III model still retained its utility in terms of accurately discovering site-invariant biomarkers. Figure 3d demonstrated that SHRED-III correctly reduced the emphasis on uncus—fusiform gyrus, a biomarker specific to UCLA, while the other models showed high salience scores.

In terms of biomarker discovery, our study revealed both site-invariant biomarkers (transverse temporal gyrus-paracentral lobule) as well as several site-specific salient FC features. These analyses provide more granular insights than existing evaluations^32,33^. Furthermore, we highlighted an issue with the current approach of biomarker discovery (represented by DNN (SL) in Fig. 3) via deep learning models, both with^32,34^ and without^14^ data harmonisation.

For site-invariant biomarkers, Fig. 3a showed the heatmap generated from a vanilla DNN model trained on the whole dataset. The vanilla DNN model highlighted transverse temporal gyrus—paracentral lobule as a site-invariant biomarker (Fig. 4 verified that it is present and salient across all sites). However, it also picked up several other features, most notably uncus—fusiform gyrus. In hypothesis 1 (Section “Analysis of salient FC features”), we posited that some of these features will in fact be biased towards the largest site. In our schizophrenia datasets, the largest site is from UCLA, which represented more than 40% of the entire aggregated dataset. From the findings in Section “Discussion”, we found that the salient feature uncus—fusiform gyrus was predominantly found in UCLA and was likely a site-specific feature. However, the SHRED variants significantly reduced the saliency score assigned to this feature, which pointed to the fact that SHRED-III can correctly exclude site-specific features while still highlighting the site-invariant ones such as transverse temporal gyrus—paracentral lobule.

For site-specific salient FC features, from the COBRE dataset, transverse temporal gyrus was noted as a prominent feature, with salient connections with six other ROIs. The transverse temporal gyrus, also known as Heschl’s gyrus, is responsible for processing incoming auditory information and it has been demonstrated that an increase in blood oxygen level–dependent occurs at the Heschl’s gyrus during episodes of auditory hallucinations^35^. For resting state fMRI, it has been found to be implicated in SZ patients too, with studies showing that SZ patients with auditory hallucinations had higher left Heschl's gyrus functional connectivity with left frontoparietal regions^36^. Among the ROIs that have salient connections with the Heschl’s gyrus, those unique to COBRE included cuneus, which was also found to have higher FC in SZ patients^37^.

From the IMH dataset, paracentral lobule has salient connections with five other ROIs. The paracentral lobule influences motor functions in the lower limbs and it was noted that components of motor abnormalities that could be caused by paracentral deficits were useful for predicting 1-year outcome in first-episode schizophrenia^38^. Furthermore, in a meta-analysis of resting state fMRI studies on schizophrenia^39^, it was noted that SZ patients have significantly lower local FC density in the paracentral lobule.

From the UCLA dataset, there are two notable site-specific salient FC features: the connection between the uncus and fusiform gyrus and between the transverse temporal gyrus and postcentral gyrus. Fusiform gyrus has long been associated with the ability to recognise faces and interpret facial expressions^40^. Past studies using traditional machine learning methods have also implicated the fusiform gyrus in schizophrenia^41^. On the other hand, the uncus is involved in olfaction, emotions and memory formation^42^ while the postcentral gyrus is involved in receiving sensory inputs and their roles in the neural basis of schizophrenia needs further evaluation.

Overall, in the existing literature, it has been demonstrated that schizophrenia patients have significant impairments in connections between various brain networks, including auditory, salience, default mode, executive control and sensorimotor networks^43^. In this study, the most salient connections involved ROIs in the auditory, visual and sensorimotor networks. In particular, a strong site-invariant biomarker in transverse temporal gyrus—paracentral lobule involves the auditory and sensorimotor networks. Each site-specific salient FC feature were shown to involve unique pairs of brain networks and the site-specific feature from COBRE (involving an across-network connection between the auditory and visual networks) have been reported in a previous study^44^, albeit not having an exact match of ROIs.

One key limitation of this study was the uncertainty with regards to salient FC features that were neither unique enough to be site-specific, nor widespread enough to be considered site-invariant. For example, the connection between the transverse temporal gyrus and posterior cingulate was highlighted as a salient feature in COBRE but also in NMorphCH (with a relatively lower score). While SHRED-III assigned a score that is in between that of transverse temporal gyrus—paracentral lobule (clearly site-invariant) and uncus—fusiform gyrus (site-specific to UCLA), it was less clear how to interpret the other salient FC features (e.g., those with dark orange/red colour on the heatmaps) that laid between these extremes. Future work could involve the design of an algorithm to identify a threshold that separates site-invariant biomarkers and site-specific salient FC features based on the saliency scores in heatmaps seen in Figs. 3 and 4.

One potential area of future work is to extend SHRED to work in a federated learning^45^ setting. Although numerous data consortiums have been formed in recent years, it is often still the case that datasets are not allowed to be shared outside of the site due to data privacy concerns. Current versions of SHRED still require pooling datasets from multiple sites together for it to produce site-invariant biomarkers. Future work could explore how site-invariant biomarkers could be arrived at without the need to pool datasets together.

Another possible future work would be to extend SHRED in terms of both data modalities and tasks. While this study was limited to static FC, dynamic FC have been shown to be effective in distinguishing SZ patients from healthy subjects and revealing SZ subtypes^46^. To allow dynamic FC data to be modelled under our framework, the architecture can be enhanced by adding recurrent neural networks (or transformers) to capture both spatial and temporal relationships^47^. To allow subtypes to be captured, a clustering module could be introduced into the auto-encoder^48^.

In summary, the neural network architecture proposed in this work (SHRED-III) could perform data harmonisation and semi-supervised learning simultaneously via an encoder-decoder-classifier architecture to deal with site differences and labelling inconsistencies even in smaller datasets. Thus, it potentially offers a scalable approach of discovering site-invariant disease biomarkers and it helps to isolate site-specific salient FC features without the need to scour each site manually (which will be increasingly unfeasible as pooled datasets and data consortia get larger). Our examination of 4 schizophrenia datasets revealed transverse temporal gyrus-paracentral lobule as a site-invariant biomarker and an example of a site-specific salient FC feature observed in our local site include multiple ROIs linked to the paracentral lobule which were not seen in other sites.

### Supplementary Information

Supplementary Information.

## Data Availability

NMorphCH and COBRE were obtained from SchizConnect http://schizconnect.org. Data from UCLA were obtained from https://openneuro.org/datasets/ds000030/versions/1.0.0.
