# Supplementary material for: Elucidating salient site-specific functional connectivity features and site-invariant biomarkers in schizophrenia via deep neural networks
Source: Sci Rep. 2023 Nov 29;13:21047. doi: 10.1038/s41598-023-48548-w (PMC10687079; doi:10.1038/s41598-023-48548-w)
Supplement: Supplementary file 1 — Supplementary Information. [file 41598_2023_48548_MOESM1_ESM.docx]

**Elucidating salient site-specific functional connectivity features and site-invariant biomarkers in schizophrenia via deep neural networks**

Yi Hao Chan, Wei Chee Yew, Qian Hui Chew, Kang Sim, Jagath C. Rajapakse

**Supplementary Tables**

|  | DNN | DNN  (DH) | **EDC  (SL)** | **EDC  (SL, DH)** | EDC  (SSL) | SHRED-II |
| --- | --- | --- | --- | --- | --- | --- |
| COBRE | 77.78  ± 2.26 | 78.52  ± 1.35 | 81.78  ± 1.12 | 82.30  ± 0.89 | 82.00  ± 0.78 | 82.15  ± 1.23 |
| IMH | 61.61  ± 4.07 | 57.96  ± 2.97 | 73.18  ± 4.76 | 71.00  ± 4.44 | 77.93  ± 4.67 | 69.96  ± 2.96 |
| NMorphCH | 66.01  ± 3.54 | 63.40  ± 4.07 | 75.33  ± 1.39 | 74.53  ± 2.08 | 75.58  ± 2.02 | 75.35  ± 1.81 |
| UCLA | 79.48  ± 1.34 | 81.05  ± 1.74 | 83.39  ± 1.12 | 83.68  ± 1.25 | 83.84  ± 1.39 | 83.74  ± 1.23 |

Table S1. Effects of data harmonisation. SHRED-II is equivalent to EDC (SSL + ComBat)

|  | SL | DH | SSL | DH + SSL | | |
| --- | --- | --- | --- | --- | --- | --- |
|  | DNN | DNN | EDC | SHRED | SHRED-II | SHRED-III |
| COBRE | 72.53 | 73.73 | 83.07 | 78.93 | 80.53 | 82.93 |
| IMH | 24.83 | 15.00 | 75.00 | 62.50 | 64.83 | 75.67 |
| NMorphCH | 36.28 | 28.00 | 72.00 | 69.43 | 68.86 | 73.14 |
| UCLA | 98.76 | 97.35 | 95.22 | 96.19 | 95.20 | 95.45 |

Table S2. Mean specificity of the 5 model architectures on individual sites.
(SL = supervised learning, DH = data harmonisation, SSL = semi-supervised learning)

|  | DNN | DNN (DH) | EDC | SHRED | SHRED-II | SHRED-III |
| --- | --- | --- | --- | --- | --- | --- |
| Whole dataset | 80.96 | 83.59 | 81.77 | 83.46 | 83.62 | 85.05 |

Table S3. Mean specificity of the 5 model architectures on the whole dataset (i.e. only SL).

|  | SL | DH | SSL | DH + SSL | | |
| --- | --- | --- | --- | --- | --- | --- |
|  | DNN | DNN | EDC | SHRED | SHRED-II | SHRED-III |
| COBRE | 84.33 | 84.50 | 80.67 | 71.83 | 84.17 | 69.33 |
| IMH | 94.50 | 97.00 | 80.50 | 83.50 | 73.00 | 82.00 |
| NMorphCH | 93.39 | 96.14 | 78.71 | 76.00 | 81.36 | 69.36 |
| UCLA | 32.80 | 41.60 | 56.30 | 53.00 | 56.00 | 56.00 |

Table S4. Mean sensitivity of the 5 model architectures on individual sites.
(SL = supervised learning, DH = data harmonisation, SSL = semi-supervised learning)

|  | DNN | DNN (DH) | EDC | SHRED | SHRED-II | SHRED-III |
| --- | --- | --- | --- | --- | --- | --- |
| Whole dataset | 70.16 | 58.45 | 69.01 | 52.58 | 60.11 | 60.73 |

Table S5. Mean sensitivity of the 5 model architectures on the whole dataset (i.e. only SL).

**Supplementary Figures**


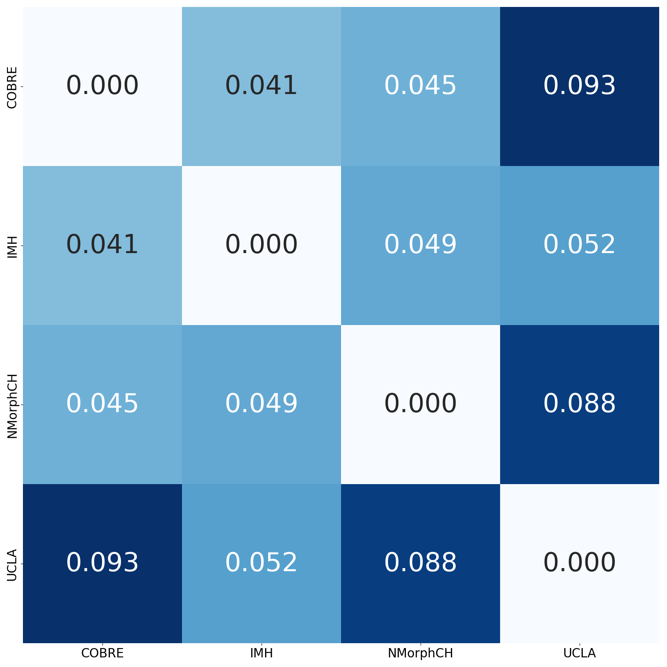

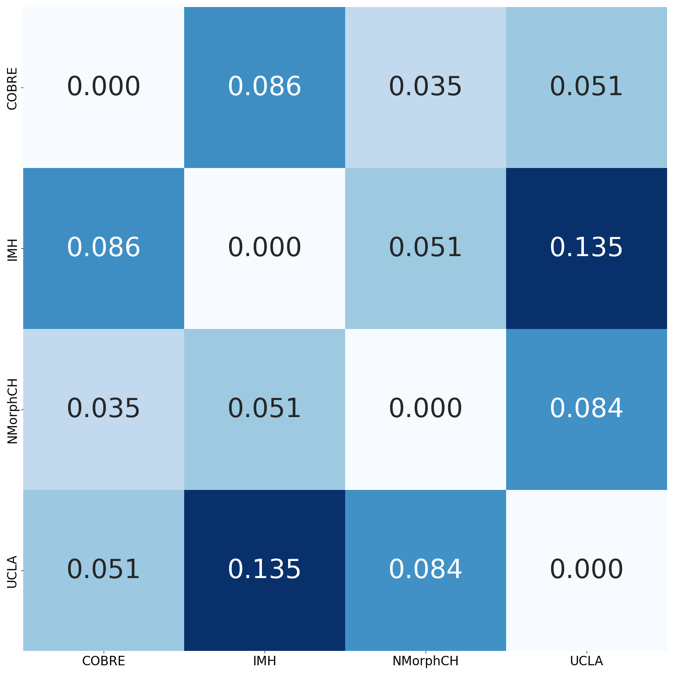

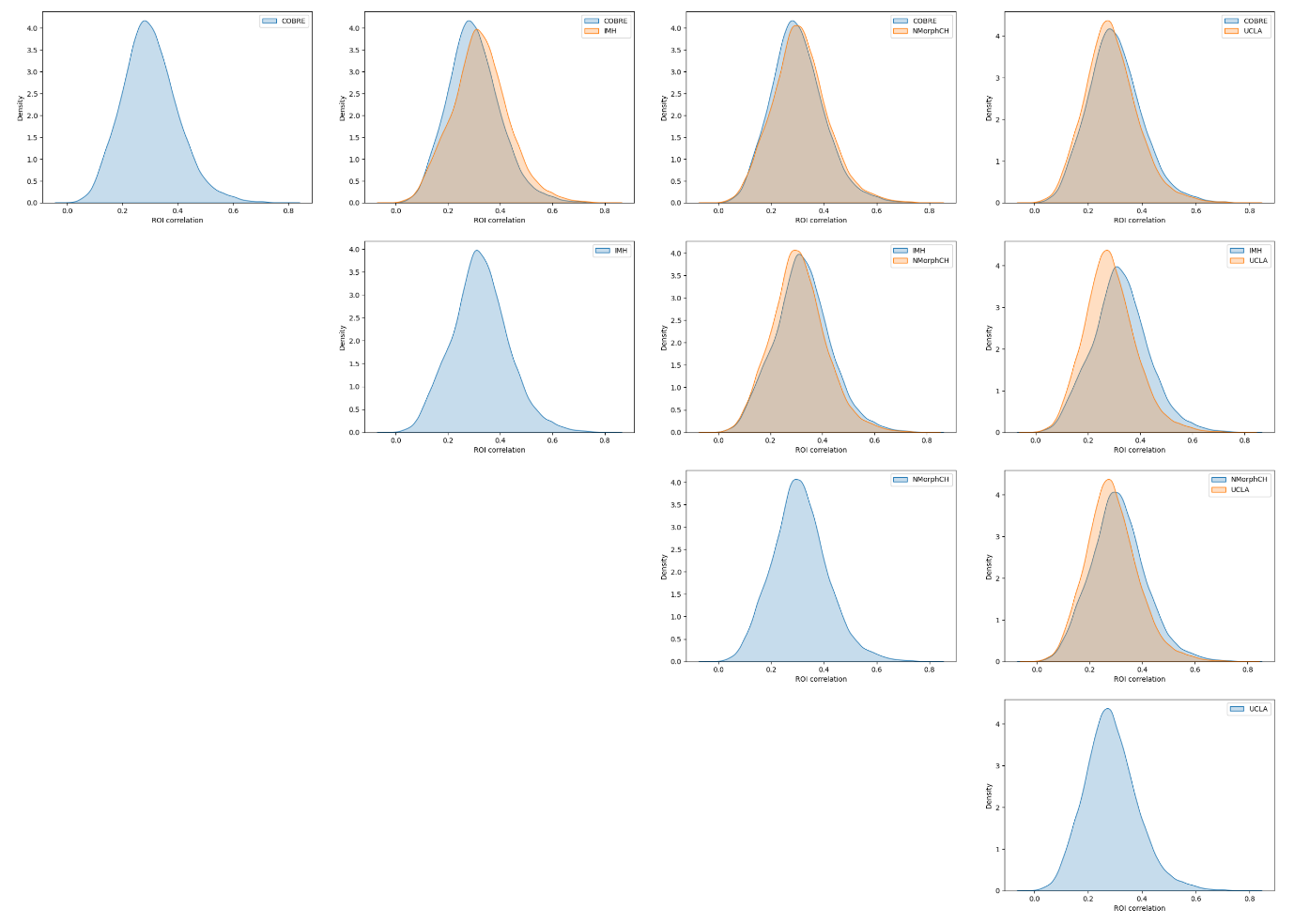


Figure S1: Hellinger distances between pairs of sites after ComBat-based harmonisation (top left) compared with SHRED-based harmonisation (top right); Kernel Distribution Estimation plots of the dataset distribution from each site after SHRED-based harmonisation (bottom)


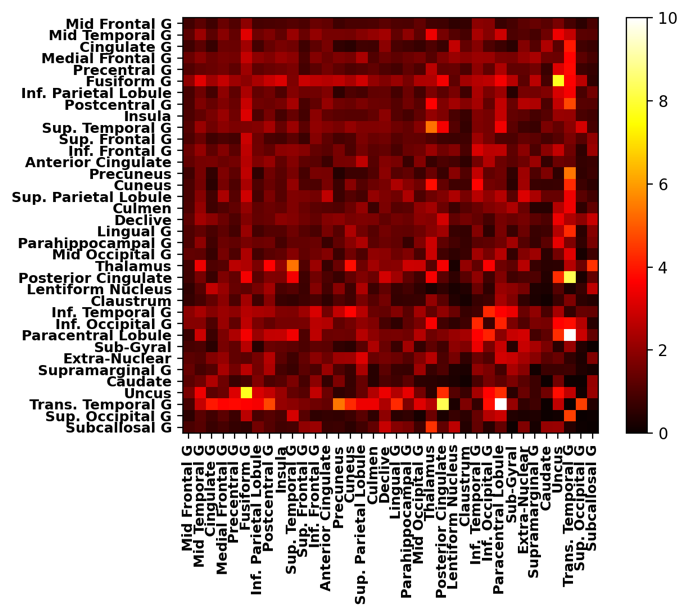


Figure S2: DNN (SL) + ComBat (whole dataset)


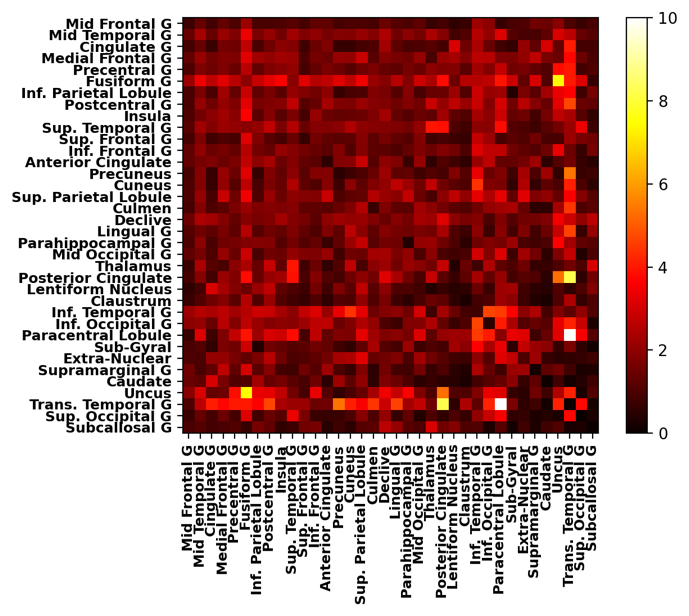

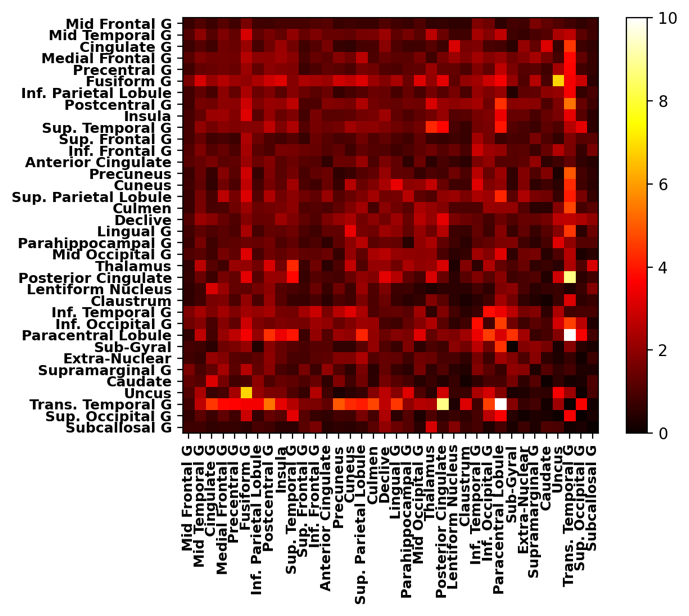


(a) DNN (b) EDC


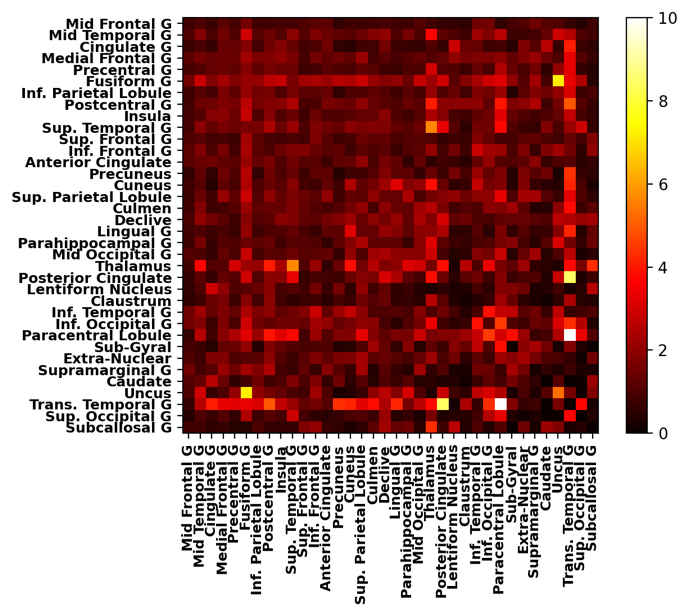

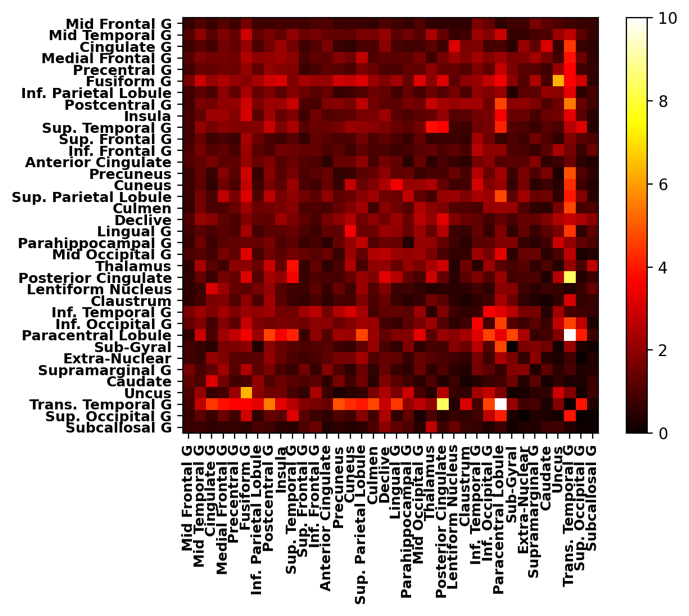


(c) SHRED-II (d) SHRED-III

Figure S3. Complete heatmaps of saliency scores from various models for the whole dataset.


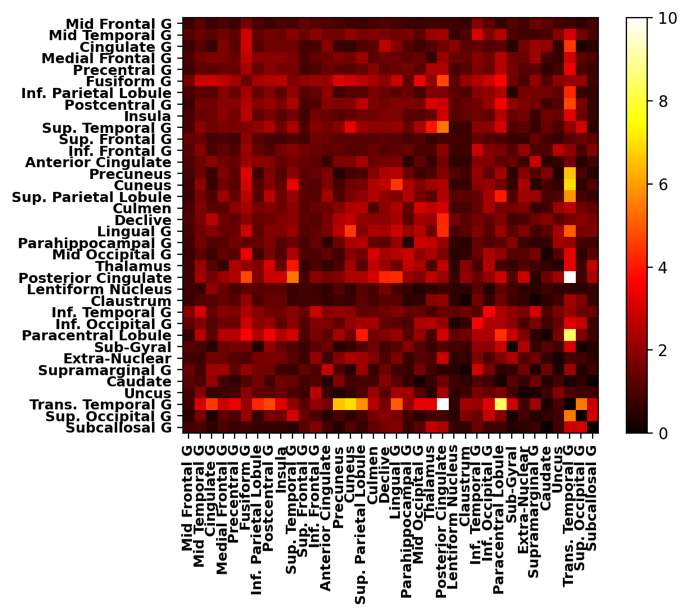

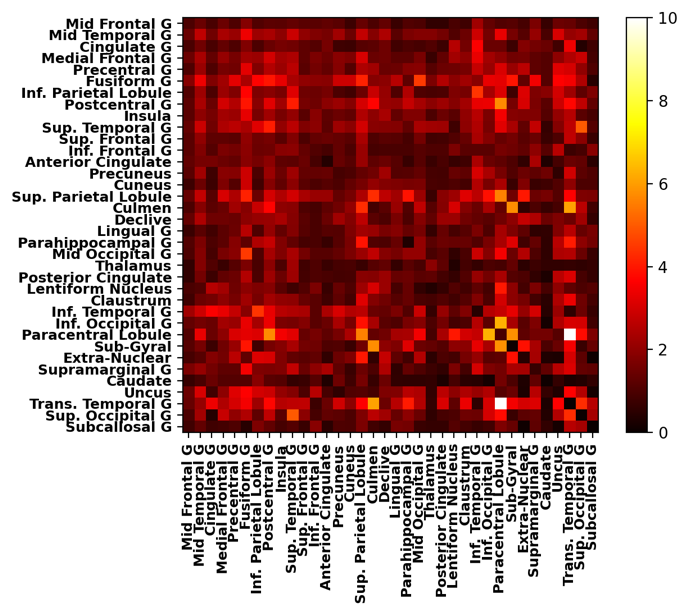


(a) COBRE (b) IMH


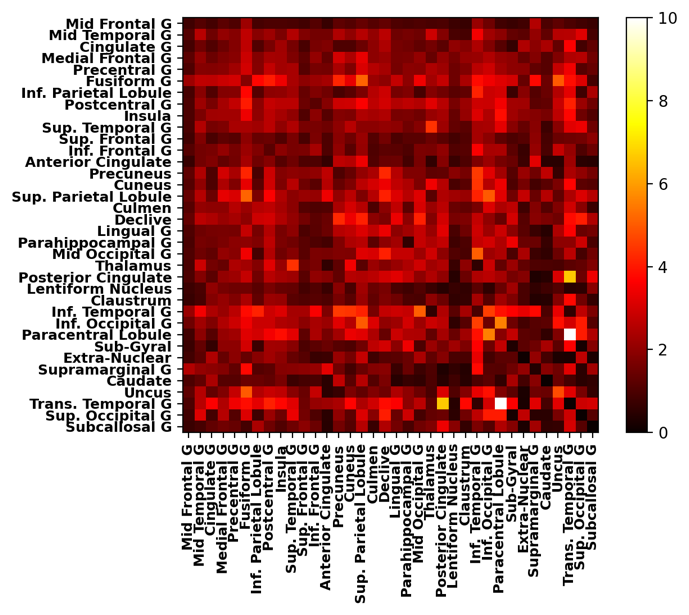

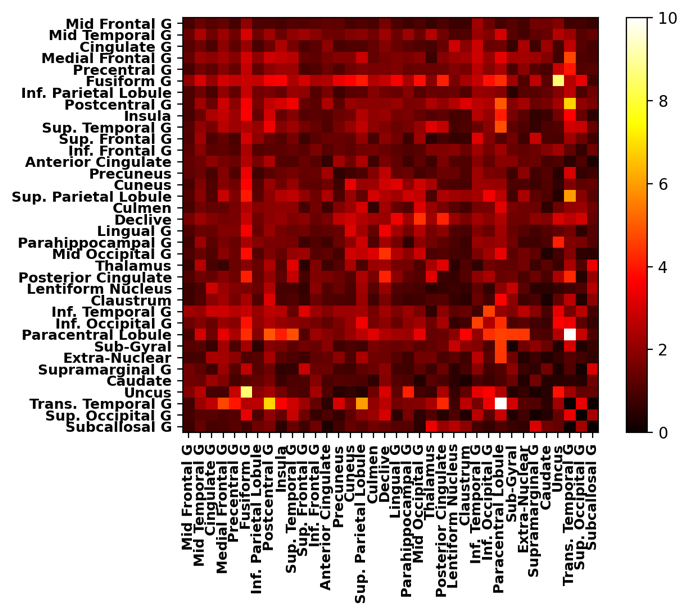


(c) NMorphCH (d) UCLA

Figure S4. Complete heatmaps of saliency scores from EDC (SL) for individual sites.


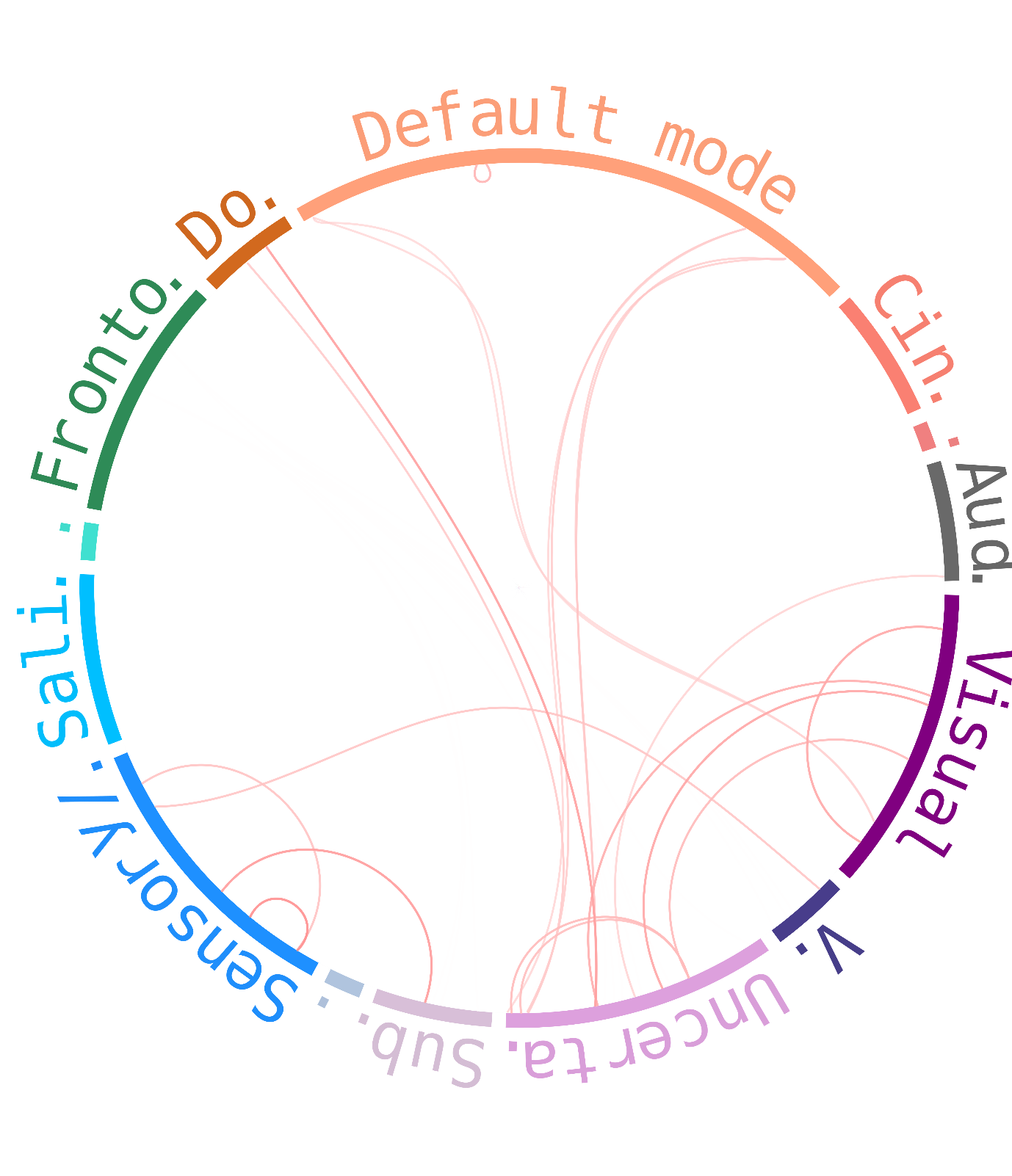


Figure S5. Chord diagram of top 0.05% salient FC values (group-level, Fisher-transformed) for SZ, with the same colour scale as Figure 5. Brain network labels as follows (anticlockwise from right): Auditory, Cerebellar, Cingulo-opercular Task Control, Default mode, Dorsal attention, Fronto-parietal Task Control, Memory retrieval, Salience, Sensory/somatomotor Hand, Sensory/somatomotor Mouth, Subcortical, Uncertain, Ventral attention, Visual.

**Supplementary Note**

**Summarised details of the 4 included datasets**

1. Center for Biomedical Research Excellence (COBRE) consists of subjects recruited from Albuquerque, New Mexico. A 3T Siemens TIM Trio scanner with a 12-channel head coil was used. During the fMRI scans, participants stared passively at a central cross. Each SZ patient (demonstrating retrospective and prospective clinical stability) completed the Structured Clinical Interview for DSM-IV Axis I Disorders, and diagnostic confirmation was made by two research psychiatrists. SZ patients with history of neurological disorders in the past year were excluded. Additional details such as exclusion criteria for healthy participants can be found in Aine et al.^41^.
2. IMH, a local dataset from Singapore. A 3T Philips Achieva scanner with an 8-channel head coil was used. For the fMRI scans, participants were instructed to keep their gaze on a central fixation cross. The eligibility of study participant was assessed via a board-certificated psychiatrist who reviewed their medical records, interviewed with their significant others, and administered the Structured Clinical Interview for DSM-IV. Participants were excluded if they had any significant history of neurological illness or if they met DSM-IV criteria for alcohol or other substance abuse. Healthy participants were excluded if they have a first-degree relative suffering from mental illness. Additional details about the IMH dataset (such as the PANSS scores) can be found in Sun et al.^42^.
3. Details about the acquisition process and patient enrolment criteria for the dataset from Neuromorphometry by Computer Algorithm Chicago (NMorphCH) are not readily available from a manuscript, but the dataset metadata revealed that a 3T Siemens TIM Trio scanner was used.
4. UCLA Consortium for Neuropsychiatric Phenomics LA5c Study (UCLA) contains scans from patients of various racial groups in the Los Angeles area. While the complete study contains patients with other neuropsychiatric disorders, only scans from healthy controls and SZ patients were used in this study. Two 3T Siemens Trio scanner were used to collect the scans. For the fMRI scan, participants were asked to keep their eyes open. DSM-IV was administered by trained interviewers and healthy participants were excluded if they have lifetime diagnoses of a range of neuropsychiatric disorders (more details can be found in Poldrack et al.^22^).

**Preprocessing details**

Results included in this manuscript come from preprocessing performed using *fMRIPrep* 21.0.1 [6,7] (RRID:SCR_016216), which is based on *Nipype* 1.6.1 [9,10].

**Anatomical data preprocessing**

A total of 1 T1-weighted (T1w) images were found within the input BIDS dataset. The T1-weighted (T1w) image was corrected for intensity non-uniformity (INU) with N4BiasFieldCorrection [17], distributed with ANTs 2.3.3 [2], and used as T1w-reference throughout the workflow. The T1w-reference was then skull-stripped with a *Nipype* implementation of the antsBrainExtraction.sh workflow (from ANTs), using OASIS30ANTs as target template. Brain tissue segmentation of cerebrospinal fluid (CSF), white-matter (WM) and gray-matter (GM) was performed on the brain-extracted T1w using fast [18] (FSL 6.0.5.1:57b01774, RRID:SCR_002823). Brain surfaces were reconstructed using recon-all [5] (FreeSurfer 6.0.1, RRID:SCR_001847), and the brain mask estimated previously was refined with a custom variation of the method to reconcile ANTs-derived and FreeSurfer-derived segmentations of the cortical gray-matter of Mindboggle [13] (RRID:SCR_002438). Volume-based spatial normalization to one standard space (MNI152NLin2009cAsym) was performed through nonlinear registration with antsRegistration (ANTs 2.3.3), using brain-extracted versions of both T1w reference and the T1w template. The following template was selected for spatial normalization: *ICBM 152 Nonlinear Asymmetrical template version 2009c* [8] (RRID:SCR_008796; TemplateFlow ID: MNI152NLin2009cAsym).

**Functional data preprocessing**

For each of the 1 BOLD run found per subject (across all tasks and sessions), the following preprocessing was performed. First, a reference volume and its skull-stripped version were generated using a custom methodology of *fMRIPrep*. Head-motion parameters with respect to the BOLD reference (transformation matrices, and six corresponding rotation and translation parameters) are estimated before any spatiotemporal filtering using mcflirt [12] (FSL 6.0.5.1:57b01774). BOLD runs were slice-time corrected to 0.974s (0.5 of slice acquisition range 0s-1.95s) using 3dTshift from AFNI [4] (RRID:SCR_005927). The BOLD time-series (including slice-timing correction when applied) were resampled onto their original, native space by applying the transforms to correct for head-motion. These resampled BOLD time-series will be referred to as *preprocessed BOLD in original space*, or just *preprocessed BOLD*. The BOLD reference was then co-registered to the T1w reference using bbregister (FreeSurfer) which implements boundary-based registration [11]. Co-registration was configured with six degrees of freedom. Several confounding time-series were calculated based on the *preprocessed BOLD*: framewise displacement (FD), DVARS and three region-wise global signals. FD was computed using two formulations following Power [15] (absolute sum of relative motions) and Jenkinson (relative root mean square displacement between affines, [12]). FD and DVARS are calculated for each functional run, both using their implementations in *Nipype* (following the definitions by [15]). The three global signals are extracted within the CSF, the WM, and the whole-brain masks. Additionally, a set of physiological regressors were extracted to allow for component-based noise correction (*CompCor*, [3]). Principal components are estimated after high-pass filtering the *preprocessed BOLD* time-series (using a discrete cosine filter with 128s cut-off) for the two *CompCor* variants: temporal (tCompCor) and anatomical (aCompCor). tCompCor components are then calculated from the top 2% variable voxels within the brain mask. For aCompCor, three probabilistic masks (CSF, WM and combined CSF+WM) are generated in anatomical space. The implementation differs from that of Behzadi et al. in that instead of eroding the masks by 2 pixels on BOLD space, the aCompCor masks are subtracted a mask of pixels that likely contain a volume fraction of GM. This mask is obtained by dilating a GM mask extracted from the FreeSurfer’s *aseg* segmentation, and it ensures components are not extracted from voxels containing a minimal fraction of GM. Finally, these masks are resampled into BOLD space and binarized by thresholding at 0.99 (as in the original implementation). Components are also calculated separately within the WM and CSF masks. For each CompCor decomposition, the *k* components with the largest singular values are retained, such that the retained components’ time series are sufficient to explain 50 percent of variance across the nuisance mask (CSF, WM, combined, or temporal). The remaining components are dropped from consideration. The head-motion estimates calculated in the correction step were also placed within the corresponding confounds file. The confound time series derived from head motion estimates and global signals were expanded with the inclusion of temporal derivatives and quadratic terms for each [16]. Frames that exceeded a threshold of 0.5 mm FD or 1.5 standardised DVARS were annotated as motion outliers. The BOLD time-series were resampled into standard space, generating a *preprocessed BOLD run in MNI152NLin2009cAsym space*. First, a reference volume and its skull-stripped version were generated using a custom methodology of *fMRIPrep*. All resamplings can be performed with *a single interpolation step* by composing all the pertinent transformations (i.e. head-motion transform matrices, susceptibility distortion correction when available, and co-registrations to anatomical and output spaces). Gridded (volumetric) resamplings were performed using antsApplyTransforms (ANTs), configured with Lanczos interpolation to minimize the smoothing effects of other kernels [14]. Non-gridded (surface) resamplings were performed using mri_vol2surf (FreeSurfer).

Many internal operations of *fMRIPrep* use *Nilearn* 0.8.1 [1] (RRID:SCR_001362), mostly within the functional processing workflow. For more details of the pipeline, see [the section corresponding to workflows in *fMRIPrep*’s documentation](https://fmriprep.readthedocs.io/en/latest/workflows.html).

**References (Preprocessing)**

1. Abraham, Alexandre, Fabian Pedregosa, Michael Eickenberg, Philippe Gervais, Andreas Mueller, Jean Kossaifi, Alexandre Gramfort, Bertrand Thirion, and Gael Varoquaux. 2014. “Machine Learning for Neuroimaging with Scikit-Learn.” *Frontiers in Neuroinformatics* 8. <https://doi.org/10.3389/fninf.2014.00014>.
2. Avants, B. B., C. L. Epstein, M. Grossman, and J. C. Gee. 2008. “Symmetric Diffeomorphic Image Registration with Cross-Correlation: Evaluating Automated Labeling of Elderly and Neurodegenerative Brain.” *Medical Image Analysis* 12 (1): 26–41. <https://doi.org/10.1016/j.media.2007.06.004>.
3. Behzadi, Yashar, Khaled Restom, Joy Liau, and Thomas T. Liu. 2007. “A Component Based Noise Correction Method (CompCor) for BOLD and Perfusion Based fMRI.” *NeuroImage* 37 (1): 90–101. <https://doi.org/10.1016/j.neuroimage.2007.04.042>.
4. Cox, Robert W., and James S. Hyde. 1997. “Software Tools for Analysis and Visualization of fMRI Data.” *NMR in Biomedicine* 10 (4-5): 171–78. [https://doi.org/10.1002/(SICI)1099-1492(199706/08)10:4/5<171::AID-NBM453>3.0.CO;2-L](https://doi.org/10.1002/(SICI)1099-1492(199706/08)10:4/5%3C171::AID-NBM453%3E3.0.CO;2-L).
5. Dale, Anders M., Bruce Fischl, and Martin I. Sereno. 1999. “Cortical Surface-Based Analysis: I. Segmentation and Surface Reconstruction.” *NeuroImage* 9 (2): 179–94. <https://doi.org/10.1006/nimg.1998.0395>.
6. Esteban, Oscar, Ross Blair, Christopher J. Markiewicz, Shoshana L. Berleant, Craig Moodie, Feilong Ma, Ayse Ilkay Isik, et al. 2018. “fMRIPrep.” *Software*. <https://doi.org/10.5281/zenodo.852659>.
7. Esteban, Oscar, Christopher Markiewicz, Ross W Blair, Craig Moodie, Ayse Ilkay Isik, Asier Erramuzpe Aliaga, James Kent, et al. 2018. “fMRIPrep: A Robust Preprocessing Pipeline for Functional MRI.” *Nature Methods*. <https://doi.org/10.1038/s41592-018-0235-4>.
8. Fonov, VS, AC Evans, RC McKinstry, CR Almli, and DL Collins. 2009. “Unbiased Nonlinear Average Age-Appropriate Brain Templates from Birth to Adulthood.” *NeuroImage* 47, Supplement 1: S102. <https://doi.org/10.1016/S1053-8119(09)70884-5>.
9. Gorgolewski, K., C. D. Burns, C. Madison, D. Clark, Y. O. Halchenko, M. L. Waskom, and S. Ghosh. 2011. “Nipype: A Flexible, Lightweight and Extensible Neuroimaging Data Processing Framework in Python.” *Frontiers in Neuroinformatics* 5: 13. <https://doi.org/10.3389/fninf.2011.00013>.
10. Gorgolewski, Krzysztof J., Oscar Esteban, Christopher J. Markiewicz, Erik Ziegler, David Gage Ellis, Michael Philipp Notter, Dorota Jarecka, et al. 2018. “Nipype.” *Software*. <https://doi.org/10.5281/zenodo.596855>.
11. Greve, Douglas N, and Bruce Fischl. 2009. “Accurate and Robust Brain Image Alignment Using Boundary-Based Registration.” *NeuroImage* 48 (1): 63–72. <https://doi.org/10.1016/j.neuroimage.2009.06.060>.
12. Jenkinson, Mark, Peter Bannister, Michael Brady, and Stephen Smith. 2002. “Improved Optimization for the Robust and Accurate Linear Registration and Motion Correction of Brain Images.” *NeuroImage* 17 (2): 825–41. <https://doi.org/10.1006/nimg.2002.1132>.
13. Klein, Arno, Satrajit S. Ghosh, Forrest S. Bao, Joachim Giard, Yrjö Häme, Eliezer Stavsky, Noah Lee, et al. 2017. “Mindboggling Morphometry of Human Brains.” *PLOS Computational Biology* 13 (2): e1005350. <https://doi.org/10.1371/journal.pcbi.1005350>.
14. Lanczos, C. 1964. “Evaluation of Noisy Data.” *Journal of the Society for Industrial and Applied Mathematics Series B Numerical Analysis* 1 (1): 76–85. <https://doi.org/10.1137/0701007>.
15. Power, Jonathan D., Anish Mitra, Timothy O. Laumann, Abraham Z. Snyder, Bradley L. Schlaggar, and Steven E. Petersen. 2014. “Methods to Detect, Characterize, and Remove Motion Artifact in Resting State fMRI.” *NeuroImage* 84 (Supplement C): 320–41. <https://doi.org/10.1016/j.neuroimage.2013.08.048>.
16. Satterthwaite, Theodore D., Mark A. Elliott, Raphael T. Gerraty, Kosha Ruparel, James Loughead, Monica E. Calkins, Simon B. Eickhoff, et al. 2013. “An improved framework for confound regression and filtering for control of motion artifact in the preprocessing of resting-state functional connectivity data.” *NeuroImage* 64 (1): 240–56. <https://doi.org/10.1016/j.neuroimage.2012.08.052>.
17. Tustison, N. J., B. B. Avants, P. A. Cook, Y. Zheng, A. Egan, P. A. Yushkevich, and J. C. Gee. 2010. “N4itk: Improved N3 Bias Correction.” *IEEE Transactions on Medical Imaging* 29 (6): 1310–20. <https://doi.org/10.1109/TMI.2010.2046908>.
18. Zhang, Y., M. Brady, and S. Smith. 2001. “Segmentation of Brain MR Images Through a Hidden Markov Random Field Model and the Expectation-Maximization Algorithm.” *IEEE Transactions on Medical Imaging* 20 (1): 45–57. <https://doi.org/10.1109/42.906424>.
